# Streptomyces antarcticus sp. nov., isolated from Horseshoe Island, Antarctica

**DOI:** 10.1099/ijsem.0.006856

**Published:** 2025-07-28

**Authors:** Sibel Melisa Sahin, Izzet Burcin Saticioglu, Muhammed Duman, Hilal Ay

**Affiliations:** 1 Department of Molecular Biology and Genetics, Faculty of Arts and Science, Yildiz Technical University, Istanbul 34220, Turkiye; 2 Department of Aquatic Animal Diseases, Faculty of Veterinary Medicine, Bursa Uludag University, Bursa 16059, Turkiye

**Keywords:** *Actinomycetota*, Antarctica, biosynthetic gene clusters, genomics, polyphasic taxonomy

## Abstract

Three novel actinobacterial strains, H27-S2^T^, H34-AA3 and H34-S5, were isolated from soil samples collected on Horseshoe Island, Antarctica, as part of an effort to discover new *Streptomyces* species with industrial and pharmaceutical relevance. A genome-based comparative analysis was conducted to characterize the strains. The pairwise comparisons of the 16S rRNA gene sequences confirmed their affiliation with the genus *Streptomyces*, with the highest sequence identity values of 99.7–99.8% with *Streptomyces avidinii* NBRC 13429^T^. However, genome-based taxonomic analyses, including digital DNA–DNA hybridization and average nucleotide identity analyses, confirmed that these strains represent a novel species within the genus *Streptomyces*. A comprehensive genome annotation revealed that the strains possess extensive biosynthetic capabilities, particularly for secondary metabolites, antimicrobial resistance mechanisms, heavy metal resistance genes and stress adaptation systems for survival in cold environments. Their metabolic potential includes aromatic compound degradation, indicating a promising role in bioremediation, while their secondary metabolite gene clusters suggest potential applications in pharmaceutical and clinical research. Given their ability to thrive in extreme Antarctic conditions, these strains may contribute to advancements in cryotechnology and enzyme production for industrial applications. Based on phylogenomic analyses, these strains represent a novel *Streptomyces* species, for which the name *Streptomyces antarcticus* sp. nov. is proposed. The type strain is H27-S2^T^ (=DSM 114983^T^=CGMCC 4.7867^T^).

## Introduction

Antarctica represents the largest unexplored habitat that remains relatively untouched. Due to its isolation from other landmasses, intense UV radiation, extreme cold, oxidative stress and microbial competition, this region presents a critical opportunity for discovering novel bioactive compounds and unique microorganisms with potential biotechnological applications. The harsh conditions of the polar regions have attracted interest in biotechnology, as microorganisms that thrive in these environments have developed unique metabolic processes that remain largely unexplored [1,6].

Actinobacteria are commonly found in diverse habitats, such as alkaline soils, sponges, sediments, hot springs, intestines and medicinal plants. Beyond their well-known role in antibiotic production, these bacteria play essential ecological roles and hold promise for agricultural and environmental applications. In recent years, research into actinobacteria has shifted towards studying specialized habitats and extreme environments [7,10].

*Streptomyces*, the largest genus within the phylum *Actinomycetota*, is ubiquitous in both aquatic and terrestrial environments and currently has 759 species with validly published names (https://lpsn.dsmz.de/genus/streptomyces; accessed on 23 June 2025). *Streptomyces* species are of immense commercial interest due to their ability to produce an extensive array of bioactive compounds, including clinically important antibiotics such as tetracyclines, aminoglycosides, macrolides, chloramphenicol, ivermectin and rifamycins. In addition to antibiotics, the members of the genus *Streptomyces* also produce valuable pharmaceutical products with broad applications, including anticancer agents, immunostimulants, immunosuppressants, antioxidants, insecticides and antiparasitic drugs [11,18]. Given the potential for discovering new bioactive compounds, significant research has focused on exploring unstudied and extreme environments for novel actinobacterial strains [19].

This study aimed to isolate novel actinobacteria from Antarctica with diverse metabolic activities. Three strains, designated H27-S2^T^, H34-AA3 and H34-S5, were obtained from two soil samples collected on Horseshoe Island, Antarctica. Whole-genome analysis, including dDDH and ANI, confirmed that these strains represent a novel species within the genus *Streptomyces*. Strain H27-S2^T^ was identified as the type strain, leading to the proposal of *Streptomyces antarcticus* sp. nov., and a comprehensive genome analysis highlighted the significant biotechnological potential of this newly characterized *Streptomyces* species.

## Methods

### Sampling and isolation of the strains

During the sixth Turkish Antarctic Expedition, two soil samples exhibiting moderate alkalinity (pH 8.0–8.4) were collected from Horseshoe Island, Antarctica, at coordinates 67° 50′ 3.4″ S 67° 13′ 41″ W and 67° 49′ 43.9″ S 67° 12′ 22.8″ W. The isolation process commenced with the aseptic grinding of the samples using a mortar and pestle, followed by serial dilution in Ringer’s solution (Oxoid). The resulting suspensions were plated onto selective media using the spread-plate technique to facilitate the isolation of actinobacteria. Incubation was carried out at 17 °C for a 30-day period. Colonies displaying firm texture, spore formation or distinct pigmentation were selected for further analysis. Strains H27-S2^T^ and H34-S5 were purified from SM3 agar (glucose 10 g l^−1^, peptone 5 g l^−1^, tryptone 3 g l^−1^ and agar 18 g l^−1^; pH 7.2), while strain H34-AA3 was obtained from Actinomycete Isolation Agar (sodium caseinate 2 g l^−1^, l-asparagine 0.1 g l^−1^, sodium propionate 4 g l^−1^, K_2_HPO_4_ 0.5 g l^−1^, MgSO_4_ 0.5 g l^−1^, FeSO_4_ 0.001 g l^−1^ and agar 18 g l^−1^; pH 8). Both media were supplemented with nystatin (25 µg ml^−1^) and cycloheximide (50 µg ml^−1^) to inhibit fungal growth. The isolates were maintained on GYM agar medium (DSMZ Medium No. 65) and NZ-Amine medium (DSMZ Medium No. 554) before being preserved in 30% (v/v) glycerol at −20 °C and −80 °C. The purified strains were then deposited in the German Collection of Microorganisms and Cell Cultures GmbH (DSMZ) and the China General Microbiological Culture Collection Center (CGMCC).

### 16S rRNA gene analysis

The strains were cultivated on NZ-Amine medium (DSMZ Medium No. 554) at 17 °C for 7 days, after which genomic DNA was extracted and purified using the PureLink^™^ Genomic DNA Isolation Kit (Invitrogen), following the manufacturer’s protocol. Amplification of the 16S rRNA gene was performed using universal primers 27F [5′-AGAGTTTGATC(AC)TGGCTCAG-3′] and 1492R [5′-ACGG(CT)TACCTTGTTACGACTT-3′] in a thermal cycler. The PCR conditions included an initial denaturation step at 98 °C for 2 min, followed by 30 cycles comprising denaturation at 98 °C for 1 min, annealing at 55 °C for 1 min 30 s and extension at 72 °C for 3 min, concluding with a final extension at 72 °C for 10 min. The extracted DNA and PCR amplicons were subjected to both quantitative and qualitative evaluation via 0.8 and 1% agarose gel electrophoresis, respectively. The PCR products were sequenced using an ABI PRISM 3730 XL automatic sequencer by Macrogen Inc. (The Netherlands) with universal primers 518F (5′-CCAGCAGCCGCGGTAATACG-3′), 800R (5′-TACCAGGGTATCTAATCC-3′) and MG5F (5′-AAACTCAAAGGAATTGACGG-3′). Sequences were assembled using ChromasPro software 1.7.6 (Technolysium Ltd.), and nearly complete sequences of 1,478 bp, 1,486 bp and 1,510 bp were obtained for strains H27-S2^T^, H34-AA3 and H34-S5, respectively. Pairwise sequence comparisons were carried out using the EzBioCloud server (https://www.ezbiocloud.net/) [20]. The 16S rRNA gene sequences of the closely related members of the genus *Streptomyces* were retrieved from the EzBioCloud and aligned using the ClustalW algorithm [21] in mega 12 v 0.10 software [22]. *Kitasatospora setae* KM-6054^T^ was used as the outgroup. Phylogenetic trees were constructed using both the neighbour-joining [23] and maximum-likelihood [24] methods, with 1,000 bootstrap replicates to assess statistical robustness [25]. Phylogenetic distances were computed based on the Tamura–Nei model [26]. The nearly complete 16S rRNA gene sequences for strains H27-S2^T^, H34-AA3 and H34-S5 were deposited in the NCBI GenBank database under accession numbers OP093992, OP093993 and OP093999, respectively.

### Genome sequencing and comparative genomics

The whole-genome sequencing was performed by MicrobesNG (Birmingham, UK) using Illumina technology. The sequencing reads in fastq format were generated with the Illumina NovaSeq 6000 (Illumina, San Diego, USA) following a 250 bp paired-end protocol and utilizing a 1,000-cycle HiSeq reagent kit. Raw reads were processed using Trimmomatic v 0.3 [27] to remove adapter sequences. Genome assembly was acquired on the KBase server (https://www.kbase.us/) [28] using the SPAdes v1.3.4 algorithm [29]. A comprehensive phylogenetic analysis of the whole genome was conducted via the TYGS web server (https://tygs.dsmz.de/) [30], employing the Genome Blast Distance Phylogeny approach with the d_4_ formula for digital DNA–DNA hybridization (dDDH). Additionally, the average nucleotide identity (ANI) values were assessed using the JSpeciesWS web server (https://jspecies.ribohost.com/jspeciesws/) [31] through both blast+ (ANIb) and MUMmer (ANIm) algorithms. Comparative ANI analyses were performed using genomic sequence data of closely related strains, which were obtained from the TYGS platform and retrieved from the NCBI database (https://www.ncbi.nlm.nih.gov/). The draft genome sequences of strains H27-S2^T^, H34-AA3 and H34-S5 have been deposited in the NCBI GenBank database under accession numbers JAPNLM000000000, JAPNLN000000000 and JAPVLP000000000, respectively.

### Functional genome analysis

The functional annotation of the bacterial genomes was carried out using the RASTtk pipeline [32] on the Rapid Annotations using Subsystems Technology (RAST) server (https://rast.nmpdr.org) [33]. The annotation identified genomic features related to resistance against toxic compounds, stress adaptation, aromatic compound metabolism, CRISPR-associated genes and prophages. To further explore the biotechnological potential of the strains, particularly in bioremediation and human health applications, and also determine the genomic features related to chemotaxonomic characteristics, a comprehensive genome analysis was performed using the BlastKOALA tool from the Kyoto Encyclopedia of Genes and Genomes (KEGG) database (https://www.kegg.jp/kegg/) [34]. The biosynthetic gene clusters (BGCs) involved in secondary metabolite production were identified using the antiSMASH web server (https://antismash.secondarymetabolites.org) [35] and compared against the MIBiG database [36]. The antimicrobial resistance-associated genes were analysed through the Antibiotic Resistant Target Seeker (ARTS) Version 2 server (https://arts.ziemertlab.com/index) [37] and the Comprehensive Antibiotic Resistance Database (CARD) (https://card.mcmaster.ca/analyze/rgi) [38,39]. The pathogenicity potential of the strains and the presence of mobile genetic elements were assessed using the PathogenFinder algorithm (https://cge.food.dtu.dk/services/PathogenFinder/) [40] and MEF (MobileElementFinder) (https://cge.food.dtu.dk/services/MobileElementFinder/) [41], respectively, from the Center for Genomic Epidemiology. The prophages were predicted using the PHASTEST (PHAge Search Tool with Enhanced Sequence Translation) web server (https://phastest.ca/) [42]. In addition, ecological distribution and habitat preference analysis based on the 16S rRNA gene sequences and genome sequence data were performed using the Galaxy Protologger server v 22.05 (http://www.protologger.de) [43].

### Phenotypic characterization

Following the 16S rRNA sequencing and whole-genome analysis, strain H27-S2^T^ was identified as a representative of its group, demonstrating strong potential to be classified as a novel species. The strain underwent extensive phenotypic characterization, which included an assessment of 80 traits encompassing carbon and nitrogen source utilization, temperature and pH tolerance, salt tolerance, biochemical properties and substrate degradation tests. Growth was evaluated on ten different media formulations, including the International *Streptomyces* Project [44] media ISP2, ISP3, ISP4, ISP5, ISP6 and ISP7, and Bennett’s agar [45], Czapek–Dox agar [46], nutrient agar (DSMZ medium no. 1) and tryptic soy agar (DSMZ medium no. 535). Growth characteristics, including substrate mycelium, aerial mycelium formation and diffusible pigment production, were systematically recorded across all media. The test strains were suspended in 2 ml of Ringer’s solution, and 200 µl of the suspension was inoculated onto appropriate culture media. The plates were then incubated at 17 °C for 14 days.

Carbon source utilization was assessed using ISP9 medium [44], while nitrogen utilization followed the methodology outlined in [47]. The tolerance tests were performed following the procedures described in [48]. Degradation assays were conducted according to the methods in [47]. Media preparation for biochemical tests followed protocols described in [49,53]. Biochemical activities, such as allantoin, arbutin and aesculin hydrolysis; urease activity; and hydrogen sulphide (H₂S) production, were examined according to [52], while nitrate reduction was evaluated based on methods described in [52,54]. All results were interpreted after a 14-day incubation at 17 °C by comparing test samples with uninoculated negative controls.

## Results and discussion

### Phenotypic characteristics of the strains

Light microscopy analysis of cells cultivated on NZ-Amine medium (DSMZ Medium No. 554) at 20 °C for 7 days reveals filamentous morphology with Gram-positive staining characteristics. Under the same conditions, colonies of strain H27-S2^T^ grown on both GYM medium (DSMZ Medium No. 65) and NZ-Amine medium display a beige to yellowish, opaque appearance with a firm substrate mycelium and smooth edges. Strain H27-S2^T^ could produce aerial mycelium on ISP2, ISP3, ISP4, Czapek–Dox, GYM agar and STMS (DSMZ Medium No. 252) media (Table 1 and Fig. S1, available in the online Supplementary Material). Both strains H34-AA3 and H34-S5 showed similar morphological characteristics with those of strain H27-S2^T^ when cultured on GYM and NZ-Amine, while displaying distinct aerial mycelium characteristics on STMS and ISP3 media (Fig. S2). Phenotypic analyses showed that strain H27-S2^T^ could utilize various substrates as sole nitrogen sources, including l-alanine, l-asparagine, glycine, l-hydroxyproline, l-histidine, l-isoleucine, l-proline, l-serine, l-cysteine, l-tyrosine and l-threonine but not l-phenylalanine or l-methionine. The strain could utilize d-glucose, d-arabinose, d-cellobiose, d-fructose, d-mannose, d-melibiose, d-raffinose, l-glutamine, dextrin and maltose as sole carbon sources but could not utilize d-adonitol, d-galactose, d-melibiose, inulin, l-rhamnose, l-sorbose, myo-inositol, sucrose or succinic acid. The strain could tolerate temperatures ranging from 10 to 28 °C (optimum at 20 °C), grow within a pH range of 4–12 (optimum at pH 7) and tolerate NaCl up to 4% (w/v) concentration. According to the degradation tests, strain H27-S2^T^ could degrade compounds such as guanine, hypoxanthine, gelatin, casein, chitin, xanthine, xylene, starch and Tween 20, but not Tween 40. Additionally, strain H27-S2^T^ could metabolize arbutin and aesculin, but it could not metabolize allantoin and urea, produce H₂S or reduce nitrate.

**Table 1.** Growth characteristics of strain H27-S2^T^ on various media. Symbols indicate relative growth levels compared to preferred medium, NZ-Amine agar

Media	Growth	Substrate mycelium colour	Aerial mycelium colour	Diffusible pigment
ISP2	+++	Grey/green	Grey/pink	−
ISP3	+++	Grey/green	Grey/pink	−
ISP4	+++	Dark cream	Grey/pink	−
ISP5	+++	Light green	−	−
ISP6	+++	Dark cream	−	−
ISP7	+++	Green	−	−
Bennett’s agar	+++	Grey/light green	−	−
Czapek–Dox agar	+	Grey	White/pink	−
Nutrient agar	+++	Dark cream	−	−
Tryptic soy agar	+++	Cream	−	−
NZ-Amine agar	+++	Beige/yellow	−	−
GYM agar	+++	Beige/yellow	Grey	−

An *in silico* comparison of the predicted phenotypic characteristics of strains H27-S2ᵀ, H34-AA3 and H34-S5 and the closely related type strains *Streptomyces subrutilus* ATCC 27467ᵀ, *Streptomyces avidinii* DSM 40526ᵀ and *Streptomyces xanthophaeus* NRRL B-5414ᵀ was conducted using the Protologger tool. All strains were predicted to possess the following metabolic and biosynthetic pathways: siroheme biosynthesis from glutamate, starch utilization, acetate production from acetyl-CoA, propionate production from propanoyl-CoA, l-glutamate production from ammonia via l-glutamine, biotin (vitamin B7) biosynthesis from pimeloyl-ACP/CoA and cobalamin (vitamin B12) biosynthesis from cobinamide. In addition, they were predicted to degrade various carbohydrates and amino acids, including starch, melibiose, fructose, mannose, aspartate, glutamate, alanine, glycine, isoleucine, proline, cysteine, glutamine, serine and 4-aminobutyrate. Furthermore, core metabolic pathways such as the *Bifidobacterium* shunt, glycolysis (pay-off phase), the oxidative and non-oxidative branches of the pentose phosphate pathway, NADH:ferredoxin oxidoreductase, the pyruvate dehydrogenase complex and the interconversion of acetate and acetyl-CoA were also predicted across all strains. Interestingly, while all strains were predicted to possess threonine degradation pathways, strains H27-S2ᵀ, H34-AA3 and H34-S5 were found to contain only the threonine degradation II pathway, in contrast to the type strains (*S. subrutilus* ATCC 27467ᵀ, *S. avidinii* DSM 40526ᵀ and *S. xanthophaeus* NRRL B-5414ᵀ), which carried the threonine degradation I pathway. The detailed differential characteristics among these strains are provided in Table S1.

### 16S rRNA gene-based phylogenetic analysis

**Fig. 1.**
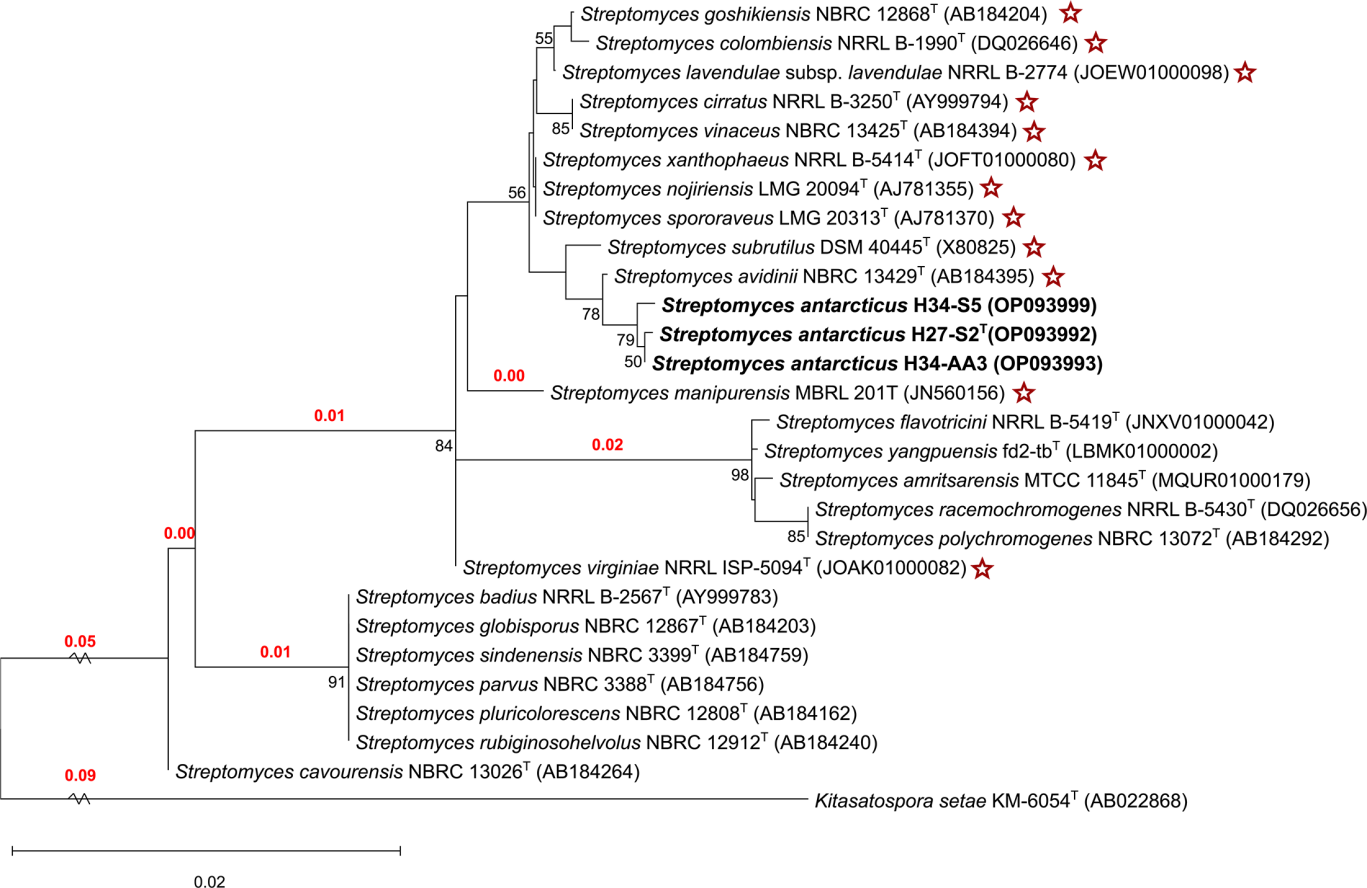
Phylogenetic tree of strains H27-S2^T^, H34-AA3 and H34-S5 and the closest type strains of the *Streptomyces* species based on 16S rRNA gene sequences using the maximum-likelihood algorithm. *K. setae* KM-6054^T^ (AB022868) is the outgroup. Bootstrap values, derived from 1,000 replications and shown when the support value is ≥50%. The scale bar corresponds to 0.02 substitutions per nucleotide position. The branch leading to the outgroup and the remaining strains at the root was shortened for clarity. Branch lengths are shown in red; branches without numerical labels have lengths of 0.00. Stars indicate the 16S rRNA sequence similarity above 98.7%.

The 16S rRNA gene sequence analysis showed that strains H27-S2^T^, H34-AA3 and H34-S5 are members of the genus *Streptomyces*. Regarding pairwise sequence comparison, both H27-S2^T^ and H34-AA3 are identical, with no difference in their 16S rRNA gene sequences, whereas both show a slight difference when compared to strain H34-S5 (Table S2). Phylogenetic analysis revealed that *S*. *avidinii* NBRC 13429^T^, *Streptomyces cirratus* NRRL B-3250^T^, *Streptomyces vinaceus* NBRC 13425^T^, *S*. *xanthophaeus* NRRL B-5414^T^, *Streptomyces nojiriensis* LMG 20094^T^, *Streptomyces spororaveus* LMG 20313^T^, *S*. *subrutilus* DSM 40445^T^, *Streptomyces lavendulae* subsp. *lavendulae* NRRL B-2774, *Streptomyces goshikiensis* NBRC 12868^T^, *Streptomyces colombiensis* NRRL B-1990^T^, *Streptomyces virginiae* NRRL ISP-5094^T^ and *Streptomyces manipurensis* MBRL 201^T^ are the closest relatives of the three strains, all showing 16S rRNA gene sequence identity values above 98.7%, exceeding the commonly accepted species demarcation threshold [55,56]. Strains H27-S2ᵀ and H34-AA3 additionally showed similarity to *Streptomyces cavourensis* NBRC 13026ᵀ, with an identity of 98.6%, placing it right at the species delineation borderline, while strain H34-S5 exhibited a notably lower similarity value, remaining well below the threshold. The phylogenetic tree based on the 16S rRNA gene sequence indicated that strains H27-S2^T^, H34-AA3 and H34-S5 formed a cluster with the type strains of *S. avidinii* NBRC 13429^T^ and *S. subrutilus* DSM 40445^T^ (Figs 1 and S3).

### Genome features and phylogenomic characteristics

The genome sequencing of strain H27-S2^T^ resulted in 89 contigs, with a coverage depth of 183-fold. The genome size was 8,504,971 bp, and the G+C content was 72.3 mol%. The N_50_ and L_50_ values were 220,250 bp and 13, respectively. The predicted genome of strain H27-S2^T^ contained 8,322 CDSs and 77 RNAs. Genome analysis identified 384 core genes, along with 31 regions related to known BGCs, 39 regions associated with known antibiotic resistance models, 43 regions exhibiting gene duplication and 254 regions that could be linked to phylogenetic relationships or horizontal gene transfer. The genomic features of strains H27-S2^T^, H34-AA3 and H34-S5 are provided in Table 2.

**Table 2.** Genomic features of the strains H27-S2^T^, H34-AA3 and H34-S5

Genomic features	H27-S2^T^	H34-AA3	H34-S5
Genome size	8.5	9.1	8.9
G+C (mol%)	72.3	71.9	72
Contigs	89	160	303
N_50_	220,250	116,243	54,499
L_50_	13	26	46
Coverage	183×	129×	43×
Completeness	99.62%	99.19%	98.24%
Contamination	0.19%	0.98%	1.07%
CDSs	8,322	9,187	9,059
RNAs	77	87	87
Core genes	384	378	378
BGCs	31	28	33
Antimicrobial resistance	39	44	44
Gene duplication	43	48	46
Horizontal gene transfer	254	212	252

**Fig. 2.**
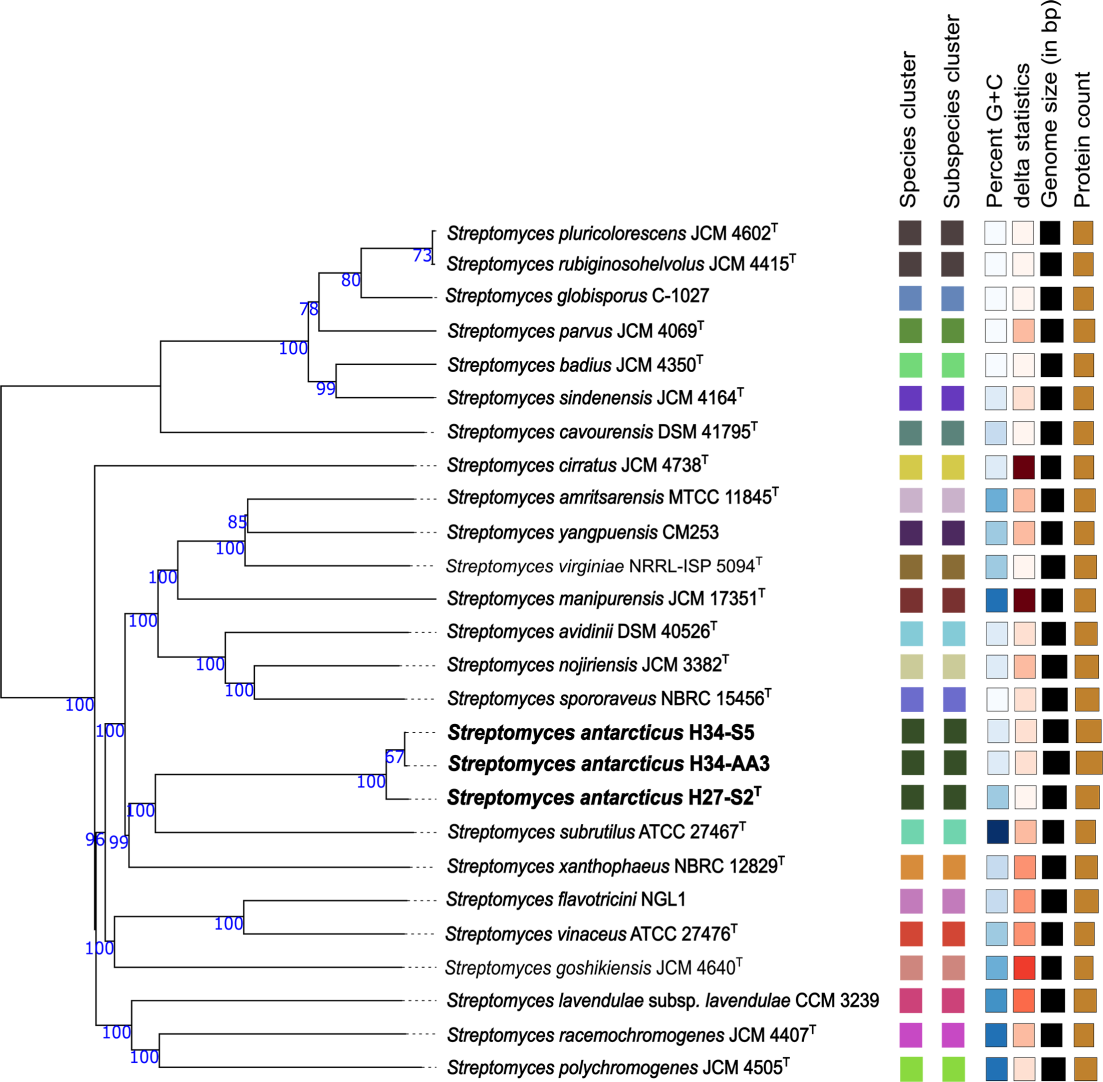
A phylogenetic tree based on the whole genome of the strains H27-S2^T^, H34-AA3 and H34-S5 was generated using the TYGS web server. Numbers in nodes represent pseudo-bootstrap values exceeding 60% across 100 replicates. On average, the phylogenetic tree is supported with a rate of 92.6%, with a delta (δ) value of 0.149. GC percentages span from 71.59% to 73.45%, genome sizes vary from 7,239,828 to 9,184,247 bp and the number of proteins falls between 6,315 and 8,624.

The whole genome-based comparative analyses revealed that strains H27-S2^T^, H34-AA3 and H34-S5 represent a novel species within the genus *Streptomyces*. In the phylogenomic tree, H27-S2^T^ clustered with strains H34-AA3 and H34-S5 while forming a clade with the type strains of *S. subrutilus* and *S. xanthophaeus* (Fig. 2). The dDDH analysis indicated that H27-S2^T^ shared 90.2% similarity with both H34-AA3 and H34-S5, while H34-AA3 and H34-S5 exhibited a 99.4% similarity, placing all three strains within the same species cluster. The ANI values calculated using blast+ (ANIb) and MUMmer (ANIm) algorithms showed that strain H27-S2^T^ shared the identity values ranging from 98.3% to 99.0% with strains H34-AA3 and H34-S5. The dDDH and ANIb/ANIm analyses identified *S. subrutilus* ATCC 27467^T^ as the closest type strain to H27-S2^T^, with respective dDDH, ANIb and ANIm values of 34.3%, 87.0% and 89.4% (Table 3). Based on established species delineation thresholds – 95–96% for ANI [57] and 70% for dDDH [58,59] – these findings confirm that strains H27-S2^T^, H34-AA3 and H34-S5 are representatives of a novel species. Consequently, strain H27-S2^T^ has been designated as the type strain of this newly identified species.

**Table 3.** Overall genomic relatedness indices between strain H27-S2^T^ and the phylogenetically close strains in the genus *Streptomyces*

GenBank assembly	**Species**	Strain	dDDH (%)	ANIb (%)	ANIm (%)
GCA_026627255	*S. antarcticus*	H27-S2^T^	100	100	100
GCA_026627185	*S. antarcticus*	H34-AA3	90.2	98.27	99.02
GCA_027157945	*S. antarcticus*	H34-S5	90.2	98.30	99.01
GCA_008704535	S. subrutilus*	ATCC 27467^T^	34.3	87.08	89.46
GCA_014648615	S. nojiriensis*	JCM 3382^T^	32.4	85.71	88.75
GCA_016755895	S. xanthophaeus*	NBRC 12829^T^	32.1	85.84	88.63
GCA_017874515	S. avidinii*	DSM 40526^T^	32.1	85.71	88.62
GCA_016755875	S. spororaveus*	NBRC 15456^T^	32.1	85.48	88.66
GCA_000720455	S. virginiae*	NRRL-ISP 5094^T^	31.7	85.45	88.44
GCA_024666385	*Streptomyces yangpuensis*	CM253	31.5	85.08	88.34
GCA_001953875	*Streptomyces amritsarensis*	MTCC 11845^T^	31.4	85.12	88.35
GCA_049570255	S. manipurensis*	JCM 17351^T^	30.3	84.65	87.98
GCA_014650555	S. goshikiensis*	JCM 4640^T^	30.0	84.44	87.91
GCA_008704935	S. vinaceus*	ATCC 27476^T^	29.9	84.59	87.89
GCA_020639365	*Streptomyces flavotricini*	NGL1	29.9	84.17	87.96
GCA_002803845	*S. lavendulae* subsp. lavendulae*	CCM 3239	29.7	84.22	87.80
GCA_039535215	*Streptomyces racemochromogenes*	JCM 4407^T^	29.3	84.01	87.68
GCA_014650775	S. cirratus*	JCM 4738^T^	29.0	83.55	87.41
GCA_039521165	*Streptomyces polychromogenes*	JCM 4505^T^	28.7	83.38	87.47
GCA_000261345	*Streptomyces globisporus*	C-1027	23.0	77.75	85.43
GCA_006788935	*S. cavourensis*	DSM 41795^T^	23.0	77.72	85.34
GCA_014649035	*Streptomyces sindenensis*	JCM 4164^T^	23.0	77.67	85.43
GCA_014649875	*Streptomyces rubiginosohelvolus*	JCM 4415^T^	22.9	77.85	85.49
GCA_014650395	*Streptomyces pluricolorescens*	JCM 4602^T^	22.9	77.63	85.51
GCA_014649415	*Streptomyces badius*	JCM 4350^T^	22.9	77.58	85.49
GCA_014648875	*Streptomyces parvus*	JCM 4069^T^	22.8	77.76	85.45

*Refers to strains exhibiting more than 98.7% sequence similarity in the 16S rRNA gene.

### Virulence analysis

The *in silico* pathogenicity analysis conducted using PathogenFinder showed that strain H27-S2^T^ has a low likelihood of being a human pathogen, with a probability of 0.167 and an input proteome coverage of 0.7%. The analysis did not identify any pathogenic families but found 54 non-pathogenic families (Table S3). Similarly, strains H34-AA3 and H34-S5 also have a low probability of being human pathogens, with probabilities of 0.172 and 0.174, respectively. Both strains contained no pathogenic protein families and included 63 to 65 non-pathogenic protein families. No prophages were identified by PHASTEST for the strains. In the RAST annotation, the genome of strain H27-S2^T^ was found to encode phage-related proteins that comprise various phage-related genes involved in structural integrity, genome integration and host interaction. It includes multiple phage tail components, such as tail fibre proteins, tail length tape-measure proteins and tail sheath proteins, which play essential roles in host recognition and infection. Several phage integrases and site-specific recombinases are present, facilitating genome integration and prophage maintenance. In addition, the dataset contains a phage endonuclease VII, crucial for DNA processing [60], and a peptidoglycan-binding endopeptidase, which likely aids in cell wall degradation during infection [61]. The presence of minor structural proteins and peptidase-associated phage proteins suggests additional roles in phage assembly and host interaction. These findings highlight the diversity of phage elements within the analysed genome and their potential roles in phage-host dynamics. Strain H34-AA3 includes several phage-related genes, including those encoding structural components such as tail fibre proteins, tail sheath proteins and major capsid proteins, which are essential for phage morphology and host recognition. It also includes multiple integrases, which facilitate phage genome integration into host DNA, and terminases involved in DNA packaging during phage assembly [62]. Additionally, several putative phage proteins, unique phage resistance genes *pglZ* and *pglY* in *Streptomyces* that are associated with phiC31 resistance [63,64] and enzymes such as endonuclease VII and peptidoglycan binding endopeptidase are present, which contribute to DNA cleavage and cell wall degradation. These genes indicate the presence of prophage elements within the genome, potentially impacting bacterial virulence, horizontal gene transfer and host interactions. The genome of strain H34-S5 also contains various phage-related genes, including multiple bacteriophage resistance genes such as *pglY* and *pglZ*. Several genes encode essential structural and functional components of bacteriophages, including tail fibre proteins, tail length tape-measure proteins, major capsid proteins and phage sheath proteins, all contributing to phage assembly and infection mechanisms. In addition, the list includes multiple terminase large subunits, which are crucial for DNA packaging within the viral capsid, as well as integrases, which facilitate phage genome integration into the host. Other notable entries are DNA helicases, DNA primases and endonucleases, indicating potential roles in phage replication and host DNA manipulation. The presence of putative prophage-related proteins and phage morphogenesis factors, such as head maturation proteases and site-specific recombinases, further suggests active or latent phage elements within the host genome. This collection of genes provides insights into phage-host interactions, resistance mechanisms and the diverse functional repertoire of bacteriophage-related elements within the genomes of the strains. Moreover, CRISPR sequences are likely to be present in the event of a prophage infection. The genome analysis of strain H34-AA3 using the RAST web server revealed the presence of three CRISPR-associated genes, including the CRISPR-associated helicase Cas3, the Cse3 family protein and the Cas5e CRISPR-associated protein. In contrast, strain H34-S5 was found to contain two CRISPR-associated genes, specifically the Cse3 family protein and the Cas5e CRISPR-associated protein. However, no CRISPR-associated genes were identified in the genome of strain H27-S2^T^. The identification of mobile genetic elements and their association with antimicrobial resistance genes and virulence factors using MEF found no mobile genetic elements in the genomes of strains H27-S2^T^, H34-AA3 and H34-S5.

### Antimicrobial resistance profiles

The antimicrobial resistance gene analysis for the three strains in the CARD database, using strict analysis and the protein homology model, identified resistance genes associated with glycopeptide resistance in the *vanW* gene cluster and small multidrug resistance antibiotic efflux pumps against disinfecting agents and antiseptics in the *qacJ* gene cluster. The *vanW* clusters were found to contribute to antibiotic target alteration, showing 30.5–32.6% identity with the matching region, while the *qacJ* gene cluster was linked to antibiotic efflux, having 44.8% identity with the matching region. In addition, the ARTS web server identified the same antimicrobial resistance models in all three strains, including aminoglycoside resistance mediated by aminoglycoside acetyltransferase, fluoroquinolone resistance involving DNA gyrase subunit B and topoisomerase IV and chloramphenicol resistance through efflux pumps, as well as beta-lactamase and metallo-beta-lactamase activity. The strains also exhibit mechanisms that affect various enzymes involved in glycolysis and gluconeogenesis, DNA replication and repair, biotin and lipoic acid binding motifs, antibiotic transport, proteasomes, amino acid biosynthesis, carboxyl transferase, pentapeptide motifs and DNA polymerase III and the beta subunit of DNA-dependent RNA polymerase (Table S4). Bacteria can develop antibiotic resistance by either mutating the target region of the WT gene or duplicating the target region. Duplication results in both an antibiotic-resistant mutant target region and a WT target region, allowing the bacteria to utilize the WT subunit in the absence of antibiotics and the mutant subunit when antibiotics are present [65]. However, especially regarding fluoroquinolone resistance, the ARTS analysis shows that strains lack duplications in the gyrase A and B regions, indicating that the organism might depend entirely on the mutant gyrase. The RAST server also predicted beta-lactamase and metallo-beta-lactamase activity, MFS efflux pumps, 3-O phosphotransferase-based chloramphenicol resistance and fluoroquinolone resistance mediated by DNA gyrase subunits A and B, further corroborating the findings from the ARTS server. Notably, the genome of strain H27-S2^T^ contains only one copy of gyrase subunit B, while the genomes of strains H34-AA3 and H34-S5 have five copies. Since the ARTS analysis shows no duplication in the gyrase genes, it can be inferred that the abundance of gene copies may be driven by the need for significant amounts of gene products to perform essential bacterial intracellular functions. In response to environmental stresses such as high temperatures or oxidative conditions, bacteria adjust the number of DNA gyrase copies to maintain genomic stability. The increased presence of gyrases helps address the heightened DNA damage and supercoiling that occur under these stress conditions. For example, thermophilic bacteria often have larger DNA gyrase proteins to handle heat-induced DNA damage. This adaptation underscores the importance of DNA gyrase in managing DNA supercoiling and repair during stress. In contrast, bacteria in more stable or controlled environments, where these stresses are less common, may not need as many gyrases [66]. This could reflect the environmental conditions in which the strains existed. The overall antimicrobial resistance profile either reflects the antibiotic-producing environment these strains previously inhabited or suggests that they themselves produce these antibiotics. Since antibiotic-producing bacteria must develop self-protection mechanisms to prevent self-destruction, the antibiotic resistance models may provide insights into the bioactive secondary metabolites encoded by these strains.

### Biosynthetic gene clusters

Regarding the secondary metabolism annotation of the RAST web server, three strains were found to encode genes responsible for auxin biosynthesis and lanthionine synthetases. Auxin biosynthesis genes comprise anthranilate phosphoribosyltransferase, phosphoribosylanthranilate isomerase, tryptophan synthase alpha chain, tryptophan synthase beta chain and aromatic-l-amino-acid decarboxylase. Auxin, a plant hormone, plays a significant role in regulating secondary metabolite production in bacteria. Its concentration can influence microbial behaviour, promoting invasive growth and enhancing resistance to various stress factors by stimulating the synthesis of lipopolysaccharides, exopolysaccharides and biofilm formation [67,68]. In bacteria, auxin biosynthesis is primarily governed by the indole-3-acetic acid (IAA) pathway, which consists of four distinct routes: (1) the indole-3-acetamide pathway, (2) the indole-3-pyruvate pathway, (3) the indole-3-acetonitrile pathway and (4) the tryptamine (TAM) pathway, which is a tryptophan-dependent route. There is also a tryptophan-independent pathway [69]. These strains likely follow the TAM pathway for auxin biosynthesis, converting chorismate to tryptophan and then to TAM, ultimately forming IAA. Given their prevalence in plant-associated environments, these strains are expected to produce auxin-related metabolites, potentially aiding plant growth through efficient auxin production.

The prediction of secondary metabolite BGCs using the antiSMASH server revealed a diverse array of biosynthetic pathways. In the genome of strain H27-S2^T^, several gene clusters displayed high similarity to known metabolites. Specifically, the melanin gene cluster demonstrated a complete (100%) match with a known melanin metabolite, which plays a key role in modulating response to UV radiation due to its antioxidant properties, radical-scavenging abilities and broad-spectrum UV absorption [70]. Similarly, a terpene gene cluster exhibited full identity with isorenieratene, a carotenoid pigment involved in light harvesting [71]. Carotenoids are essential due to their unique structural and chemical properties, contributing to antioxidant defence, photoprotection and functioning as precursors to vitamin A while enhancing immune responses. Moreover, they are extensively utilized as natural food colourants, cosmetic and pharmaceutical additives and feed supplements for improving pigmentation in aquaculture. Their potent antioxidant and photoprotective characteristics also position them as promising candidates for potential therapeutic use in cancer treatment and neurodegenerative disease prevention [72,74]. A T3PKS gene cluster displayed a perfect match with alkylresorcinol, a metabolite influencing various physiological and pathological processes, including immune system function, metabolic regulation, cell signalling and gene expression [75]. Alkylresorcinols are proposed as potential adjuvant agents for anticancer and antimicrobial therapies [76,77]. Furthermore, a NAPAA gene cluster perfectly matches ε-poly-l-lysine, a naturally occurring poly(amino acid) known for its strong antimicrobial activity, making it widely applicable in the food and pharmaceutical industries [78]. An NRPS gene cluster was found to be identical to antipain, a reversible protease inhibitor derived from actinomycetes that specifically inhibits serine and cysteine proteases, such as papain, trypsin and plasmin [79]. Due to its protease inhibitory activity, antipain holds potential for therapeutic applications in anti-inflammatory, antimicrobial and anticancer treatments, as protease inhibitors have been extensively studied for their diverse biological effects [80,82]. In addition, certain gene clusters demonstrated slightly lower but still significant similarity to known metabolites. A T2PKS gene cluster exhibited 81% similarity to gilvocarcin V, a compound known for its strong bactericidal, virucidal, cytotoxic and antitumour properties [83]. In addition, an NRPS gene cluster demonstrated a 96% similarity with the gene clusters of JBIR-126 (tambromycin), a cytotoxic nonribosomal peptide [84]. Furthermore, a hybrid NRPS and T1PKS gene cluster showed 92% similarity to miharamycins A and B, a group of nucleoside antibiotics [85]. Meanwhile, a T1PKS gene cluster displayed 77% similarity to 4-Z-annimycin, a metabolite that inhibits the sporulation of various actinobacterial genera [86]. These metabolites are particularly noteworthy due to their recognized biotechnological significance and potential industrial and pharmaceutical applications. Some gene clusters showed moderate similarity to known compounds. For example, a butyrolactone gene cluster shared 55% similarity with neocarzinostatin, an antitumour antibiotic. A T2PKS gene cluster matched a spore pigment at 66%, while an NRP-metallophore gene cluster displayed 55% similarity to a siderophore called peucechelin [87]. In addition, an NRPS gene cluster showed 72% similarity to netropsin, a metabolite with antibiotic, antiviral and antitumour properties [88,90]. Furthermore, an aminoglycoside/aminocyclitol and terpene gene cluster exhibited 55% similarity to streptomycin, while another cluster showed 61% similarity to hopene. Although these similarities are moderate, they suggest that these gene clusters may produce biosynthetic metabolites with characteristics similar to the known compounds. For instance, the aminoglycoside/aminocyclitol and terpene gene cluster might encode an antibiotic with structural or functional similarities to streptomycin. Lastly, several BGCs showed low similarity (below 25%) or no match with known metabolites, indicating the potential presence of novel biosynthetic pathways that may encode previously undiscovered metabolites.

The genome annotation using the KEGG database revealed the biochemical pathway of strains H27-S2^T^, H34-AA3 and H34-S5 related to streptomycin biosynthesis. It demonstrated that the strains possess an almost complete set of enzymes required for streptomycin production, reinforcing their antibiotic potential (Fig. S4). The genomes of strains H27-S2^T^ and H34-AA3 encode gene clusters showing a complete match to ε-poly-l-lysine and a moderate match to peucechelin gene clusters. In contrast to the genome of strain H27-S2^T^, strains H34-AA3 and H34-S5 encode a BGC identical to the ectoine gene cluster in addition to gene clusters showing moderate similarity to the gene clusters of massinidine, an antiplasmoidal alkaloid [91], and neocarzinostatin, an antitumour antibiotic [92]. Ectoine is a bacterial extremophile compound that helps protect proteins and biological membranes from damage caused by extreme conditions such as high salinity, drought, radiation, pH fluctuations and temperature variations. It has several biotechnological and biomedical applications, including acting as a protein and DNA protectant, membrane modulator, cryoprotectant and wastewater treatment, as well as managing respiratory and hypersensitivity diseases [92,95]. The presence of ectoine suggests that these strains have the capacity to survive in extreme environments, which aligns with the oligotrophic conditions, UV radiation, extreme cold and drought present in Antarctica, where the strains were originally isolated. Lastly, similar to the genome of strain H27-S2^T^, strain H34-S5 encodes a gene cluster exhibiting a complete match to melanin pigment. Further details on the BGCs of these strains and their comparisons to the MIBiG database can be found in Tables S5, S6 and S7.

### Genes related to other biotechnological applications

The stress response analysis conducted on the RAST web server showed that the strains harbour multiple heat shock proteins (HSPs), including the GroES, GroEL and GrpE, which aid in protein folding and stabilization under stress [96]. Additionally, they contain cold shock proteins (CSPs) from the CSP family, which enable the strains to survive in cold conditions by stabilizing RNA and other cellular components [97]. The strains also encode alkaline shock proteins, potentially involved in responding to alkaline stress [98], as well as ribosome-associated HSPs that contribute to protein synthesis and maintenance during stressful conditions [99,100]. These proteins are crucial for the strain’s ability to withstand environmental stress, such as temperature changes and other challenging conditions. All isolates have also shown the presence of type I antifreeze protein, indicating their cold adaptation mechanisms. The stress response mechanisms identified in these strains hold significant potential for biotechnological applications. These properties could be harnessed to produce cold-active enzymes, particularly valuable in industrial and pharmaceutical processes requiring low-temperature catalysis. Moreover, their resilience to extreme conditions suggests potential use in cryopreservation and bioremediation, particularly in environments with alkaline conditions. Furthermore, these strains could serve as biocatalysts, facilitating protein synthesis under stressful environmental conditions, thereby enhancing their applicability in various industrial and biotechnological processes.

The bioremediation potential of the strains was assessed through an analysis of aromatic compound metabolism using the RAST web server, with the results presented in Table 4. In addition, the KEGG database provided critical insights into the pathways involved in xenobiotic biodegradation. Notably, strain H27-S2^T^ appears to encode ten key enzymes responsible for the degradation of fluorouracil, a widely used chemotherapy drug, as well as the immunosuppressant azathioprine and the chemotherapy agent 6-mercaptopurine (Fig. S5). This enzymatic capability suggests potential applications in bioremediation, particularly in the removal of pharmaceutical contaminants from hospital wastewater, thereby mitigating environmental pollution and drug toxicity. Moreover, the presence of these metabolic pathways may offer valuable insights into chemotherapy resistance, as gut microbiota harbouring such strains could potentially contribute to drug inactivation, reducing therapeutic efficacy [101]. In addition to their pharmaceutical degradation potential, strains H34-AA3 and H34-S5 exhibit genetic capacity for xylene and benzoate degradation, highlighting their potential use in the breakdown of industrial pollutants, thereby reinforcing their relevance in bioremediation applications.

**Table 4.** Distribution of mechanisms related to the metabolism of aromatic compounds in *Streptomyces* isolates, revealed by the RAST web server

Metabolism of aromatic compounds	H27-S2^T^	H34-AA3	H34-S2
Gentisate degradation	+	+	+
Homogenization pathway of aromatic compound degradation	+	+	+
*p*-Hydroxybenzoate degradation	−	+	+
Salicylate and gentisate catabolism	+	+	+
Quinate degradation	+	+	+

Furthermore, the KEGG pathway mapping revealed that strain H27-S2^T^ encodes key enzymes involved in glutamate metabolism, including glutaminase, an important enzyme in synaptic network, which catalyses the hydrolysis of glutamine into glutamate and ammonia [102]; glutamate decarboxylase, which facilitates the conversion of glutamate into γ-aminobutyric acid (GABA) [103]; and glutamine synthetase, which enables the synthesis of glutamine from glutamate and ammonia [104]. The presence of these enzymes suggests that this strain may have potential applications in the biosynthesis of neuroactive compounds, particularly in the production of GABA, a key neurotransmitter with significant implications in neuropharmacology.

The resistance mechanisms have substantial biotechnological potential, offering valuable insights for the development of new drug therapies and bioremediation techniques. The resistance to heavy metals and antibiotics observed in the strains is important in biotechnology (Table S8). These resistance mechanisms indicate the strains’ survival ability in environments contaminated with toxic substances, such as heavy metals (arsenic, cobalt, copper, mercury and chromium) and antibiotics. This capability is valuable for bioremediation processes, where strains can be used to clean up polluted environments by detoxifying heavy metals or breaking down harmful compounds. In addition, their multidrug resistance might be useful in developing novel strategies for overcoming antibiotic resistance in medical applications. The strains' ability to resist oxidative stress and toxic substances could also make them candidates for industrial applications where they can function in harsh conditions, enhancing the efficiency and sustainability of biotechnological processes.

The genomic features related to the resistance to glyoxalase/bleomycin, oxytetracycline, daunorubicin/doxorubicin and tunicamycin demonstrate the significant potential of the strains for therapeutic and industrial applications. Glyoxalase enzymes play a role in detoxifying reactive species that cause DNA damage [105], and bleomycin is an anticancer drug [106]. The resistance to glyoxalase/bleomycin suggests that these strains could be beneficial in drug development or in environments where DNA damage must be managed. This resistance could also contribute to research on the mechanisms of cancer therapy resistance. Oxytetracycline is a broad-spectrum antibiotic, and resistance to it might indicate that these strains are capable of surviving in environments where antibiotics are present. Such resistance could be especially useful in agricultural biotechnology, particularly in settings where antibiotics are commonly used or in research focused on understanding antibiotic resistance mechanisms [107]. Daunorubicin and doxorubicin are potent chemotherapeutic agents [108,109], and resistance to them in the genomes of the strains could aid in understanding cancer resistance mechanisms and help develop new strategies for overcoming resistance to chemotherapy. Finally, tunicamycin is a glycosylation inhibitor affecting cell wall biosynthesis [110,111]. Resistance to tunicamycin in the strains could provide valuable insights into the processes of protein glycosylation and cell wall biosynthesis, offering the potential for biotechnological advancements, particularly in areas that require manipulation of protein folding or cell wall synthesis.

### Bioinformatic prediction of chemotaxonomy

Isoprenoid analysis of strains H27-S2^T^, H34-AA3 and H34-S5 using the KEGG database showed that the strains are capable of synthesizing menaquinones, with geranylgeranyl diphosphate serving as the isoprenoid quinone precursor (Fig. S6) via the ubiquinone and other terpenoid-quinone biosynthesis pathway (map 00130) and the terpenoid backbone biosynthesis pathway (map 00900). Diagnostic amino acid analysis of the lysine biosynthesis pathway (map 00300) identified ll-diaminopimelic acid and meso-diaminopimelic acid (Fig. S7). The polar lipid profile revealed the presence of phosphatidylethanolamine and phosphatidylserine in the glycerophospholipid metabolism pathway (map 00564) (Fig. S8). Regarding whole-cell sugars, the strains are likely to contain glucose, fructose and galactose, as UDP-glucose, UDP-galactose and fructose-6-phosphate synthesis pathways are present in the amino sugar and nucleotide sugar metabolism pathway (map 00520) (Fig. S9).

### Ecological distribution and habitat preferences

The Protologger tool was utilized to determine the prevalence and average relative abundance of the strains across 1,000 samples spanning 19 distinct environments. The genomes of strains H27-S2^T^, H34-AA3 and H34-S5 were compared against a comprehensive database of over 50,000 metagenome-assembled genomes (MAGs) from various environmental sources. The Protologger analysis indicated that no MAGs matched the genomes of the studied strains.

**Fig. 3.**
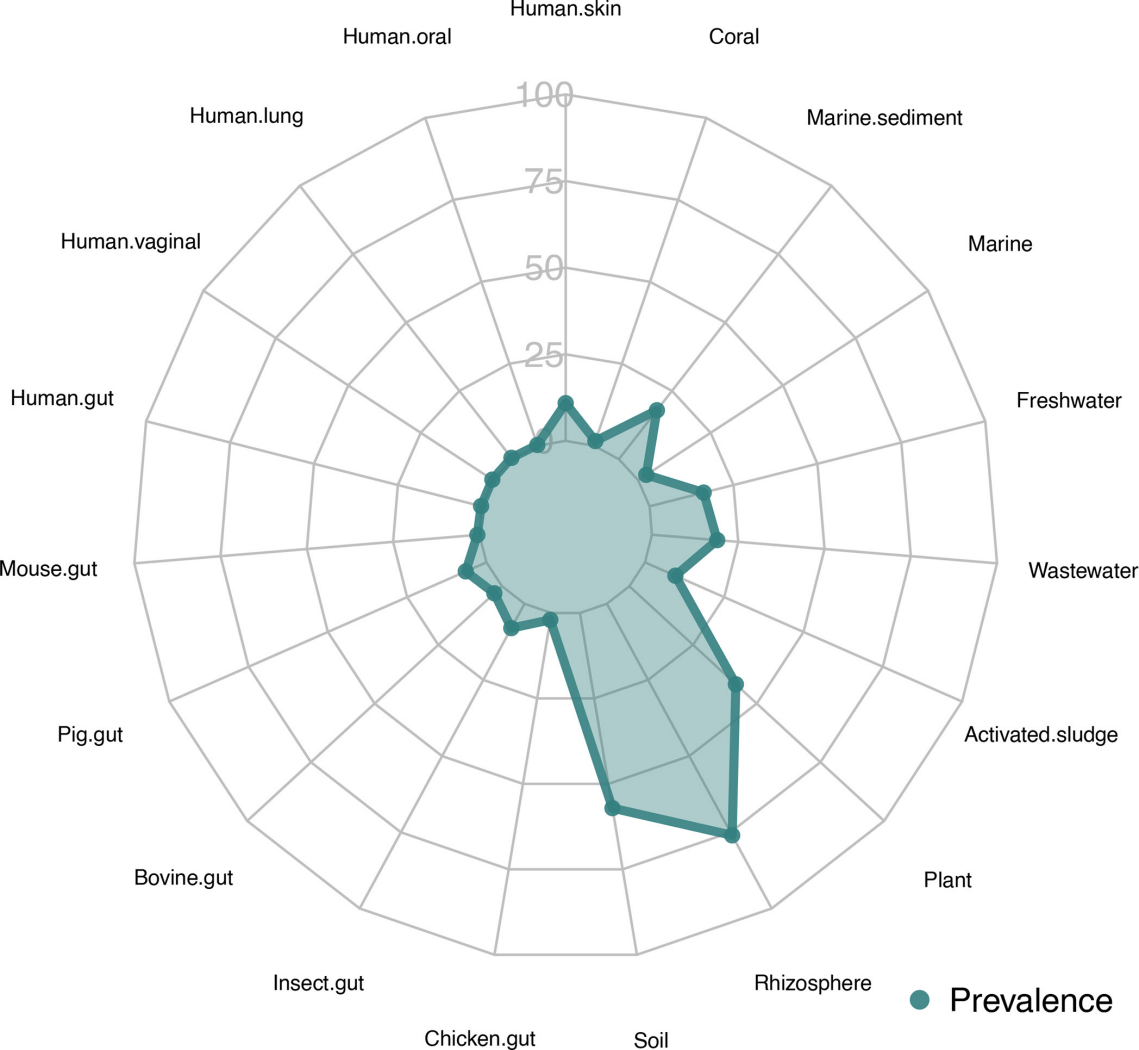
Spider plot showing the habitat distribution patterns of strain H27-S2^T^ across 19 distinct habitats using the Protologger tool.

Strain H27-S2^T^ exhibited the highest environmental preference in rhizosphere (76.0%), soil (57.1%) and plant-associated (41.8%) habitats. In contrast, its presence was observed at lower frequencies in wastewater, marine sediment, freshwater, human skin, activated sludge, insect gut, pig gut, bovine gut, marine, chicken gut, coral, mouse gut, human lung, human vaginal, human gut and human oral environments, as detailed in Table 5 and Fig. 3. Strains H34-AA3 and H34-S5 also demonstrated similar ecological distribution patterns.

**Table 5.** A highlight of ecological preferences and distribution patterns of strains H27-S2^T^, H34-AA3 and H34-S5 across 19 distinct habitats by the Protologger platform

	H27-S2^T^ and H34-AA3			H34-S5		
**Habitats**	**DR (%)**	**MRA (%)**	**SD (%)**	**DR (%)**	**MRA (%)**	**SD (%)**
Activated sludge	9.60	0.01	0.01	6.40	0.01	0.01
Bovine gut	3.00	0.05	0.12	3.00	0.05	0.12
Chicken gut	2.00	0.03	0.05	2.00	0.03	0.05
Coral	1.40	0.02	0.05	1.40	0.02	0.05
Freshwater	16.10	0.07	0.29	15.40	0.07	0.29
Human gut	0.20	0.03	0.02	0.20	0.03	0.02
Human lung	0.50	0.03	0.05	0.50	0.03	0.05
Human oral	0.20	0.02	0.01	0.20	0.02	0.01
Human skin	10.90	0.14	0.37	3.60	0.23	0.40
Human vaginal	0.30	0.00	0.00	0.20	0.00	0.00
Insect gut	8.00	0.07	0.29	7.00	0.07	0.31
Marine	2.80	0.04	0.15	2.00	0.05	0.18
Marine sediment	17.90	0.03	0.12	17.50	0.02	0.12
Mouse gut	0.60	0.02	0.02	0.50	0.02	0.02
Pig gut	6.50	0.09	0.31	5.70	0.10	0.33
Plant	41.80	2.45	5.45	41.10	2.23	5.01
Rhizosphere	76.00	0.85	1.34	74.90	0.85	1.34
Soil	57.10	0.54	2.17	51.60	0.58	2.28
Wastewater	18.90	0.13	0.70	11.90	0.14	0.40

DR, detection ratio; MRA, mean relative abundance.

## Conclusions

*Streptomyces* species are renowned for their immense biotechnological significance, particularly in the production of antibiotics, enzymes and bioactive secondary metabolites. Their metabolic versatility and ability to thrive in diverse environments make them invaluable in pharmaceutical, agricultural and industrial applications. In addition to their well-established role in natural product discovery, *Streptomyces* strains have recently gained attention for their potential in bioremediation and the bioconversion of industrial waste.

In this study, three novel Antarctic *Streptomyces* strains, collectively designated as *S. antarcticus*, have been characterized, revealing their extensive biosynthetic and metabolic capacities. The detailed analysis of the strains’ secondary metabolite gene clusters (BGCs) highlighted their impressive similarity to several known metabolites, including melanin, isorenieratene, alkylresorcinol and antipain, as well as compounds with therapeutic potential such as gilvocarcin V and JBIR-126. The identified BGCs are associated with a broad spectrum of bioactive secondary metabolites that have significant applications in drug discovery, agriculture, bioremediation and industrial biotechnology. These findings suggest that these strains possess the genetic machinery to produce metabolites with diverse bioactivities, including antimicrobial, anticancer and neuroprotective effects. Furthermore, their ability to degrade pharmaceutical compounds such as fluorouracil, azathioprine and 6-mercaptopurine highlights their possible application in remediating hospital wastewater and mitigating drug toxicity.

In addition, their cold-adaptive stress responses further enhance their industrial relevance, as they could serve as biocatalysts in low-temperature enzyme production and cryopreservation applications. The adaptation of these micro-organisms to extreme cold suggests the presence of unique enzymes and regulatory mechanisms, which could lead to groundbreaking applications in biotechnology. As research into extremophilic micro-organisms expands, these newly characterized *Streptomyces* strains may pave the way for the discovery of novel bioactive molecules, enhanced industrial biocatalysts and more efficient bioremediation strategies, ultimately broadening the scope of their biotechnological applications.

In conclusion, the findings from this study underscore the significant biotechnological potential of these strains in producing valuable metabolites with broad applications in medicine, agriculture and industry. Identifying novel biosynthetic pathways and metabolites provides exciting prospects for future research aimed at unlocking the full potential of these micro-organisms for therapeutic, agricultural and industrial use. By studying these known and novel gene clusters and understanding the underlying genetic mechanisms, it may be possible to optimize production systems for these metabolites, thereby advancing microbial biotechnology and offering innovative solutions for medicine and industry.

## Description of *Streptomyces antarcticus* sp. nov.

*Streptomyces antarcticus* (ant.arc’ti.cus. L. masc. adj. *antarcticus*, southern, of the Antarctic, pertaining to Antarctica, the geographical origin from which the type strain was first isolated).

Aerobic, Gram-positive and filamentous actinobacterium producing beige-coloured colonies on NZ-Amine agar medium and grey/pink aerial mycelia on ISP2, ISP3 and ISP4 agar media. Good growth occurs on ISP2, ISP3, ISP4, ISP5, ISP6, ISP7, Bennett’s, tryptic soy and nutrient agar media, while moderate growth occurs on Czapek–Dox agar medium. Growth occurs at 10–28 °C (optimum 20 °C), at pH 5.0–12.0 (optimum pH 7.0) and in the presence of 0–10% (w/v) NaCl (optimum 0%). Utilizes d-glucose, d-arabinose, d-cellobiose, d-fructose, d-mannose, d-melibiose, d-raffinose, l-glutamine, dextrin and maltose as sole carbon sources but not d-adonitol, d-galactose, d-melibiose, inulin, l-rhamnose, l-sorbose, myo-inositol, sucrose or succinic acid. Utilize l-alanine, l-asparagine, glycine, l-hydroxyproline, l-histidine, l-isoleucine, l-proline, l-serine, l-cysteine, l-tyrosine and l-threonine but not l-phenylalanine and l-methionine. Degrades guanine, hypoxanthine, gelatin, casein, chitin, xanthine, xylene, starch and Tween 20 but not Tween 40. Tests positive for hydrolysis of arbutin and aesculin butnegative for hydrolysis of allantoin, urea, and H₂S production and nitrate reduction.

The type strain H27-S2^T^ (=DSM 114983^T^=CGMCC 4.7867^T^) was isolated from a soil sample collected in Horseshoe Island, Antarctica. The genome size of the type strain is 8.5 Mbp, and the DNA G+C content is 72.3 mol%. The GenBank accession numbers assigned to the 16S rRNA gene sequence and the draft genome sequence of strain H27-S2^T^ are OP093992 and JAPNLM000000000, respectively.

## Supplementary material

10.1099/ijsem.0.006856 Uncited Supplementary Material 1.

## Abbreviations

ANI: average nucleotide identity
ARTS: Antibiotic Resistant Target Seeker
BGCs: biosynthetic gene clusters
CARD: Comprehensive Antibiotic Resistance Database
CGMCC: China General Microbiological Culture Collection Center
CSPs: cold shock proteins
dDDH: digital DNA–DNA hybridization
GABA: γ-aminobutyric acid
H₂S: hydrogen sulphide
HSPs: heat shock proteins
IAA: indole-3-acetic acid
KEGG: Kyoto Encyclopedia of Genes and Genomes
MAGs: metagenome-assembled genomes
MobileElementFinder: MEF
PHASTEST: PHAge Search Tool with Enhanced Sequence Translation
RAST: Rapid Annotations using Subsystems Technology
TAM: tryptamine
WT: wild-type
