# Supplementary material for: Streptomyces antarcticus sp. nov., isolated from Horseshoe Island, Antarctica
Source: Int J Syst Evol Microbiol. 2025 Jul 28;75(7):006856. doi: 10.1099/ijsem.0.006856 (PMC12451607; doi:10.1099/ijsem.0.006856)
Supplement: Uncited Supplementary Material 1. [file ijsem-75-06856-s001.pdf]

Streptomyces antarcticus sp. nov., isolated from Horseshoe Island, Antarctica

Sibel Melisa Sahin[1], Izzet Burcin Saticioglu[2], Muhammed Duman[2], Hilal Ay[1*]

[1]Yildiz Technical University, Faculty of Arts and Science, Department of Molecular Biology and Genetics, Istanbul 34220, Turkiye

[2]Department of Aquatic Animal Diseases, Faculty of Veterinary Medicine, Bursa Uludag University, Bursa 16059, Turkiye

*Corresponding author:

E-mail address: hilal.ay@yildiz.edu.tr

Department of Molecular Biology and Genetics, Faculty of Arts and Science, Yildiz Technical University, 34220, Istanbul, Turkiye

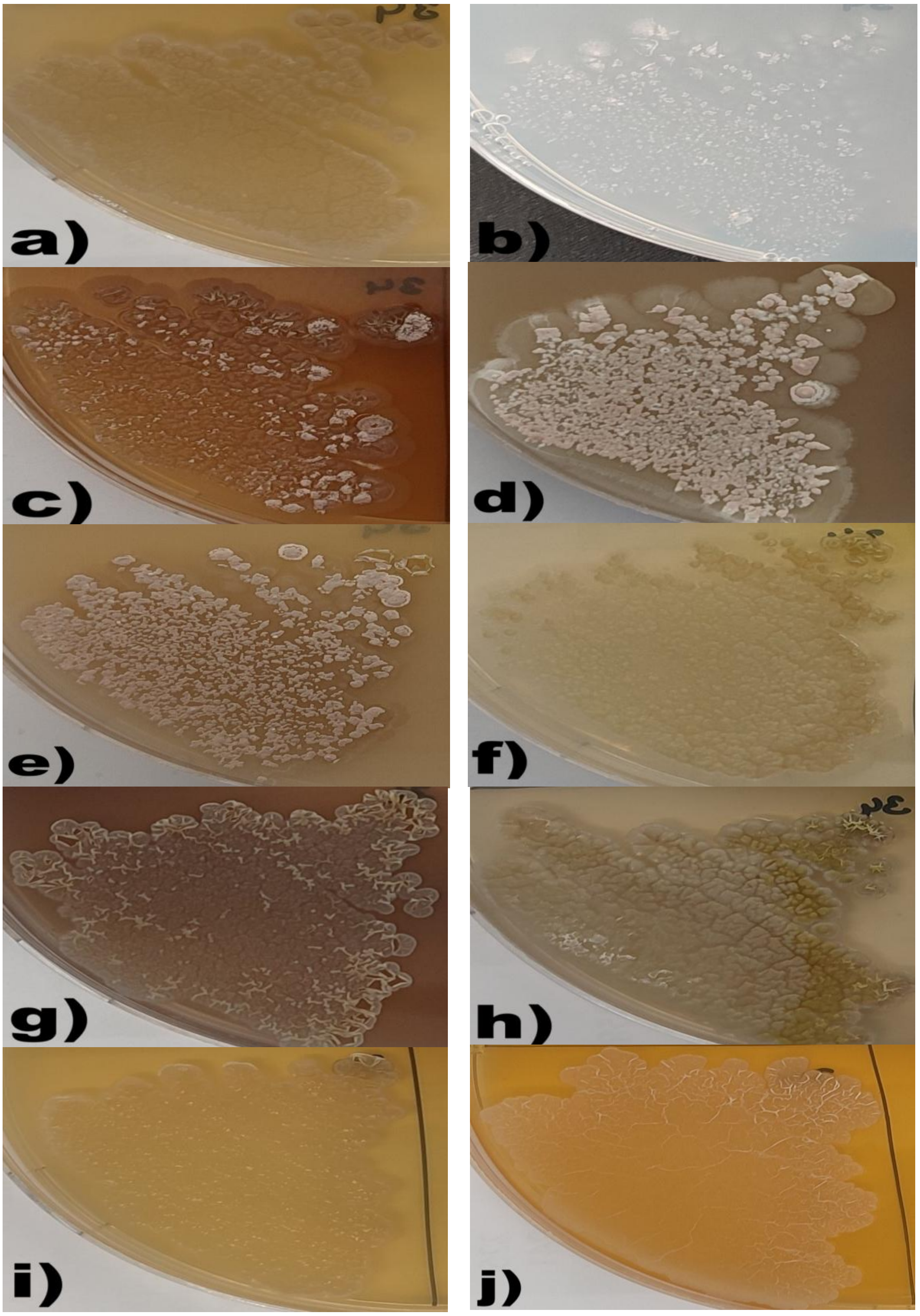

Figure S1. Growth characteristics of strain H27-S2^T on agar media after incubation at 17°C for 14 days. **a)** Bennet's Agar, **b)** Czapek's Dox Agar, **c)** ISP2, **d)** ISP3, **e)** ISP4, **f)** ISP5, **g)** ISP6, **h)** ISP7, **i)** Nutrient Agar, **j)** Tryptic Soy Agar

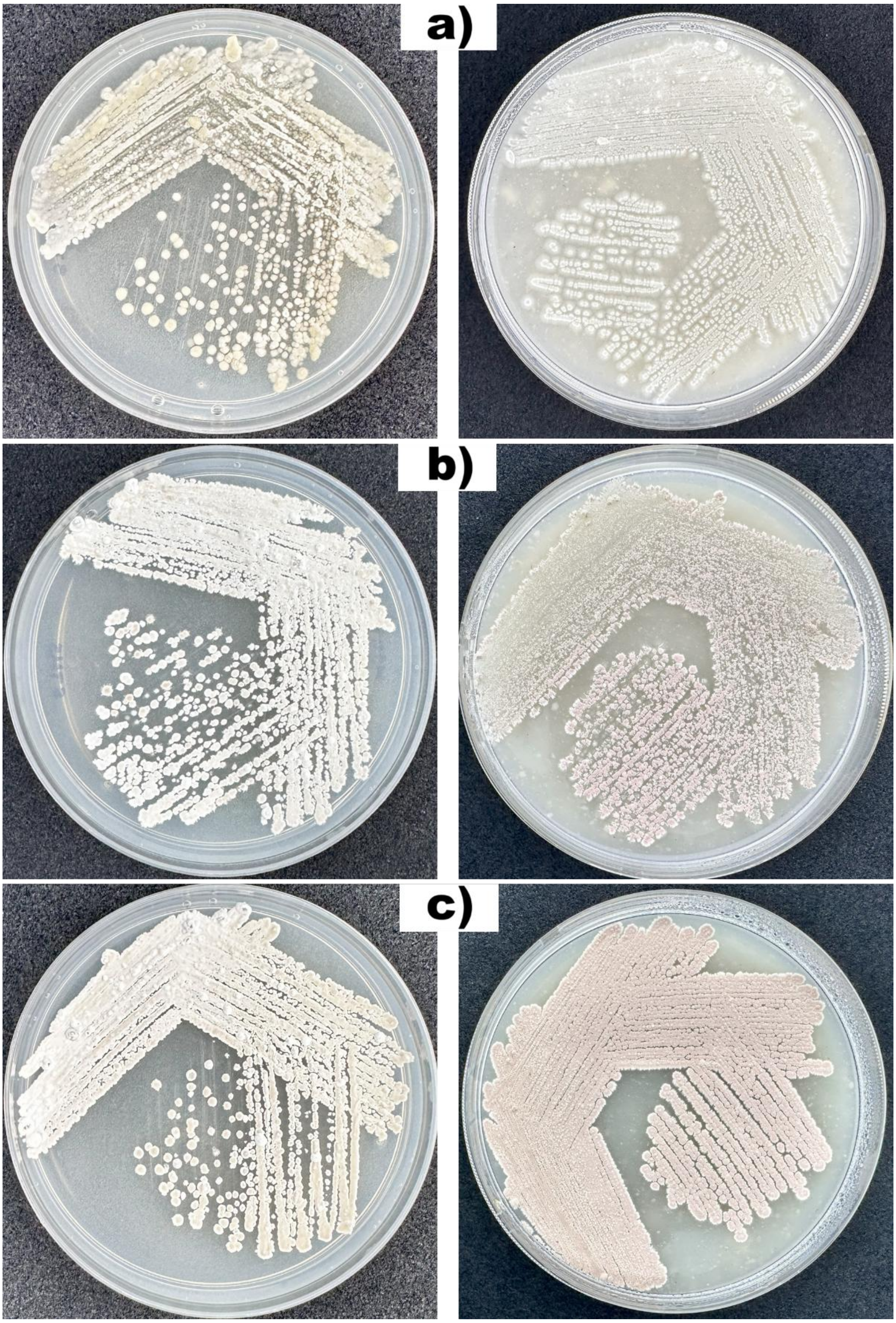

Figure S2. Aerial mycelium characteristics of strains H27-S2^{T}, H34-AA3 and H34-S5 on STMS and ISP3 agar media. **a)** H27-S2^{T} on STMS (left) and ISP3 (right), **b)** H34-AA3 on STMS (left) and ISP3 (right), **c)** H34-S5 on STMS (left) and ISP3 (right).

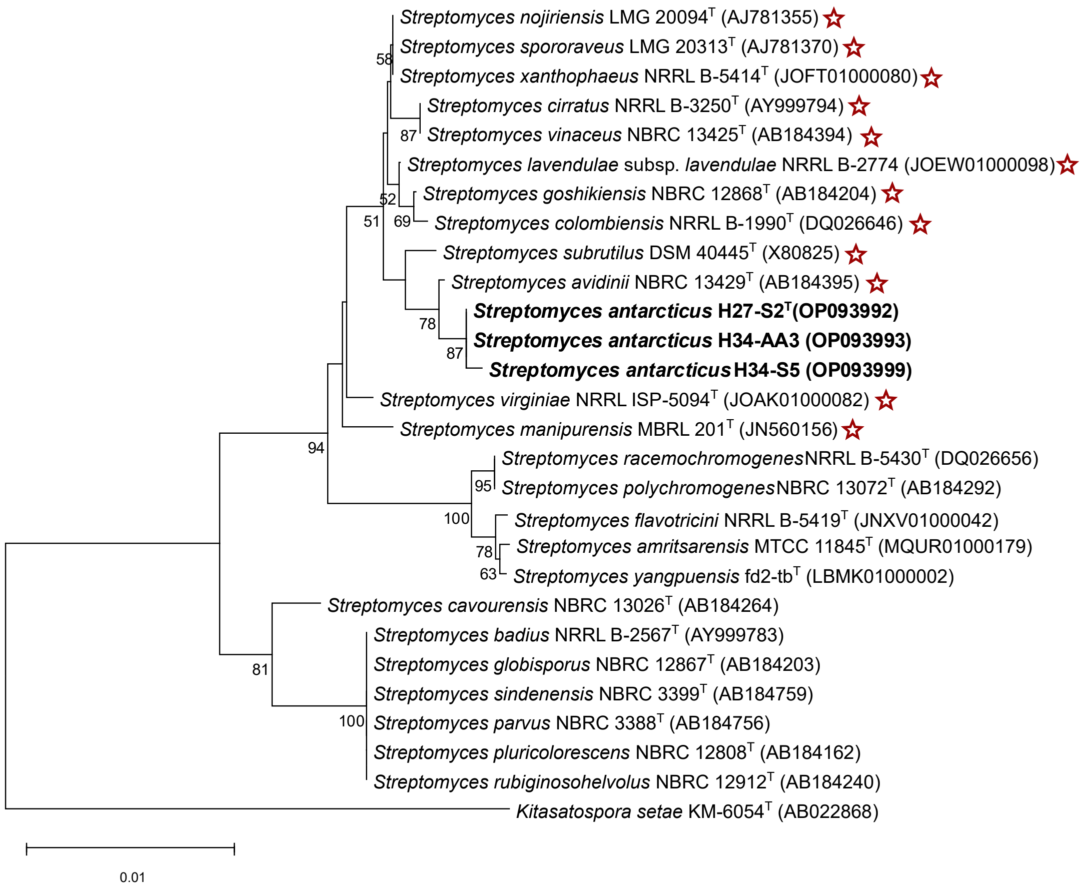

Figure S3. A phylogenetic tree of strains H27-S2^T, H34-AA3 and H34-S5 and the closely related type strains of the *Streptomyces* species, based on the 16S rRNA gene sequences using the neighbor-joining algorithm. *Kitasatospora setae* KM-6054^T (AB022868) is the outgroup. Bootstrap values, derived from 1,000 replications and exceeding 50%, are indicated. The scale bar corresponds to 0.01 substitutions per nucleotide position. * Stars indicate the 16S rRNA sequence similarity above 98.7%.

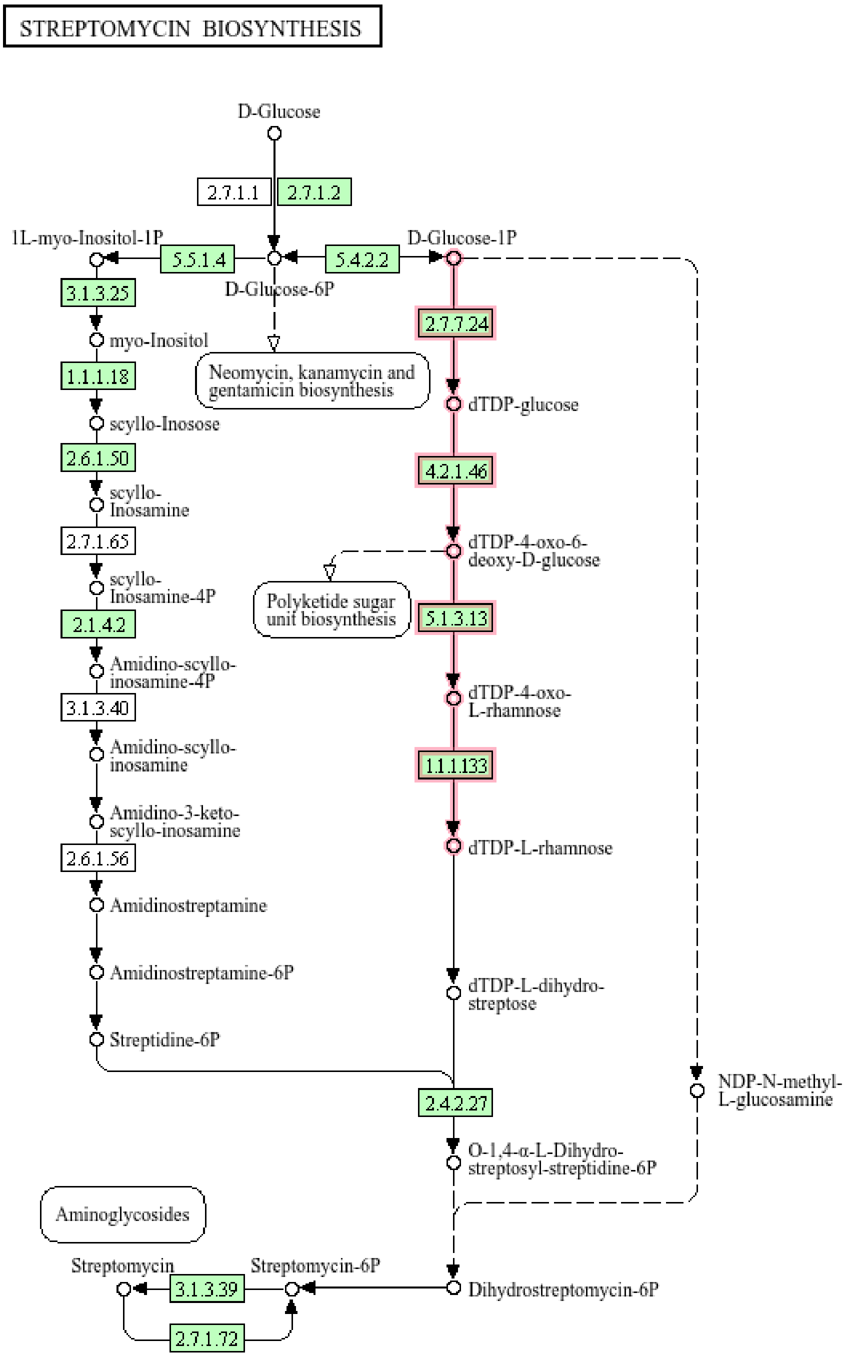

Figure S4. Biochemical pathway of enzymes for streptomycin biosynthesis encoded by the genomes of strains H27-S2^{T}, H34-AA3, H34-S5, revealed by KEGG database (KEGG Pathway: 00521).

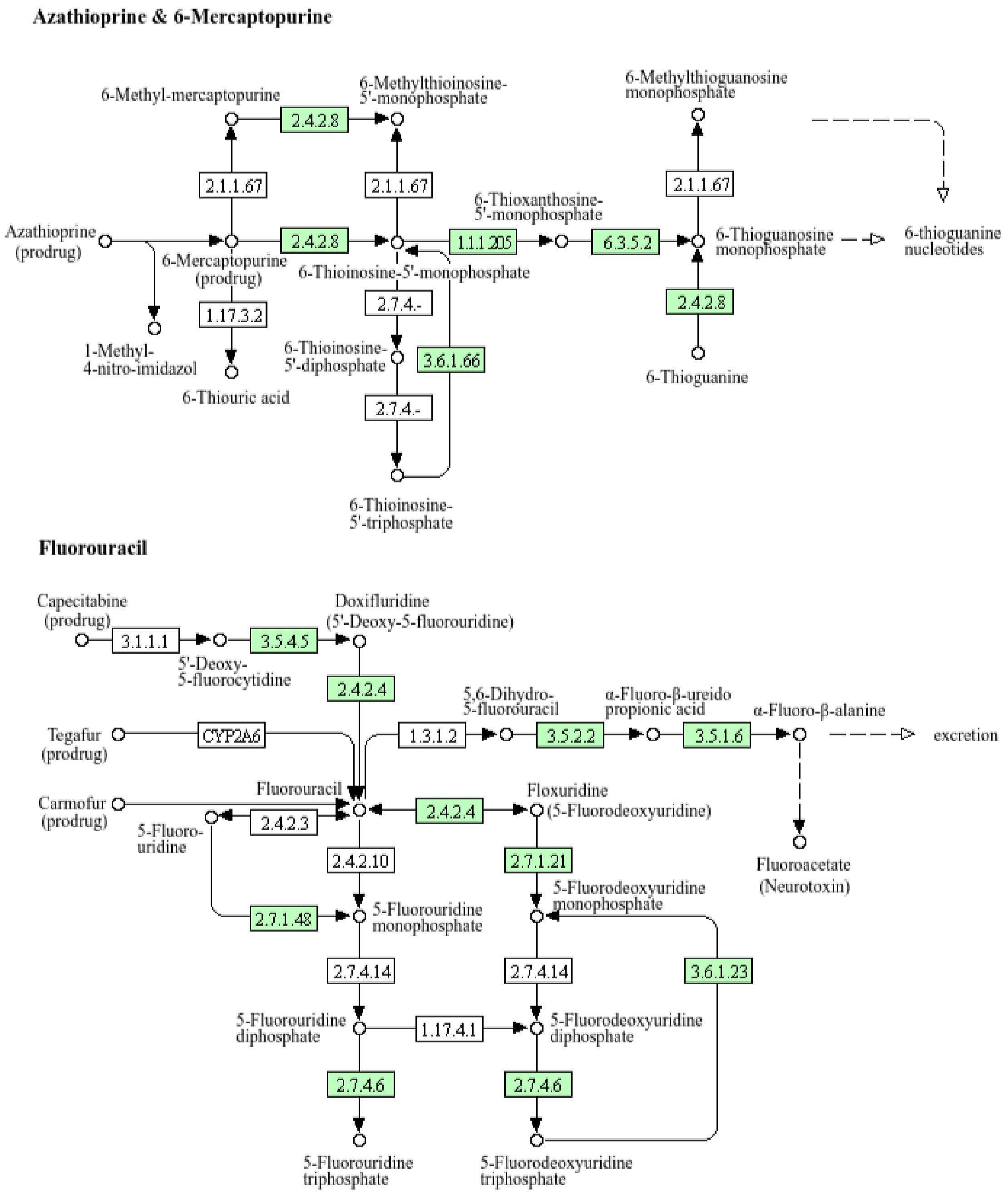

Figure S5. Biochemical pathway of enzymes for the degradation of fluorouracil and azathioprine & 6-mercaptopurine encoded by the genome of strain H27-S2^{T} , revealed by KEGG database (KEGG Pathway: 00983).

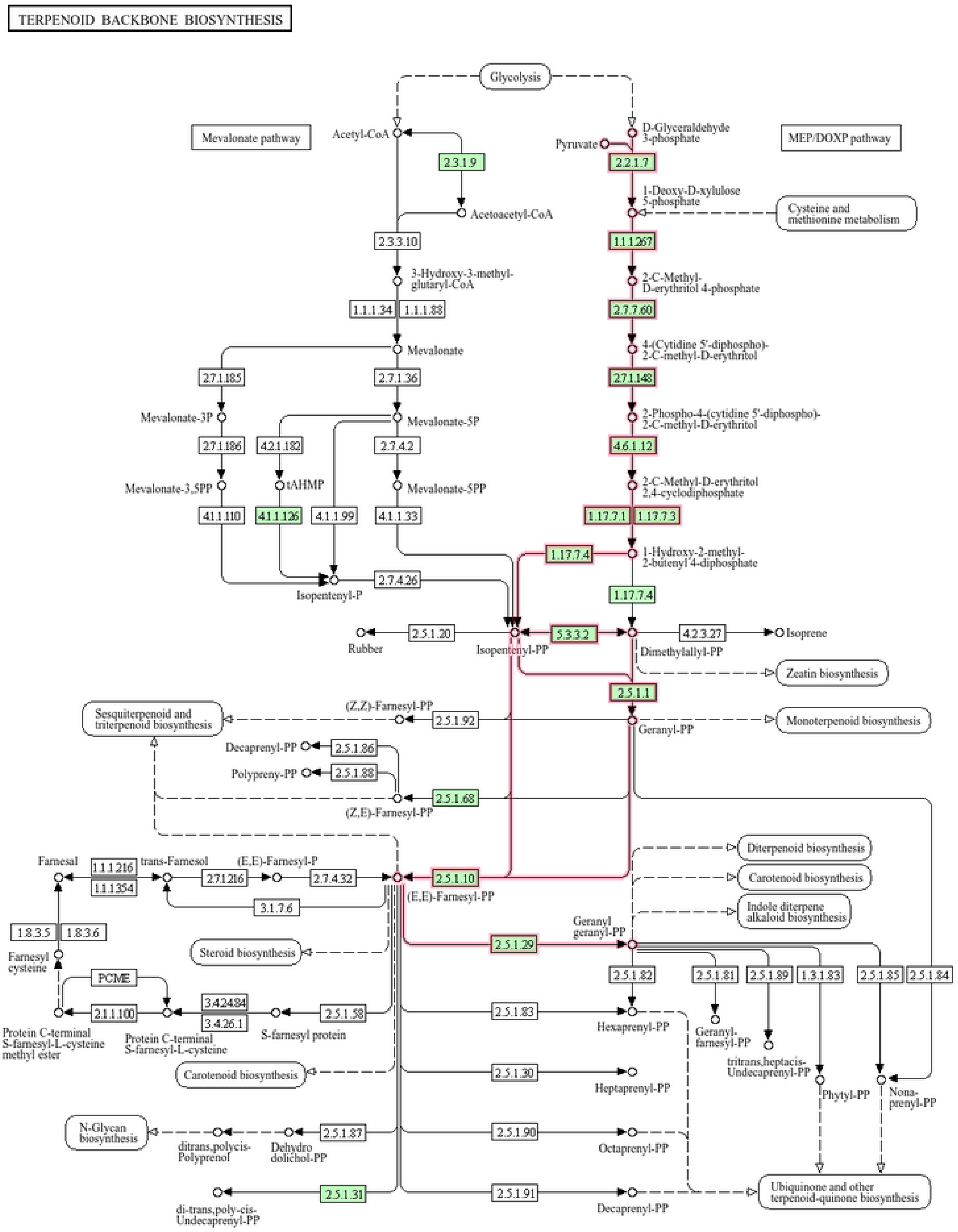

Figure S6. Biochemical pathway of enzymes and products in isoprenoid quinone synthesis encoded by the genome of strain H27-S2^{T} revealed by KEGG database (KEGG Pathway: 00900).

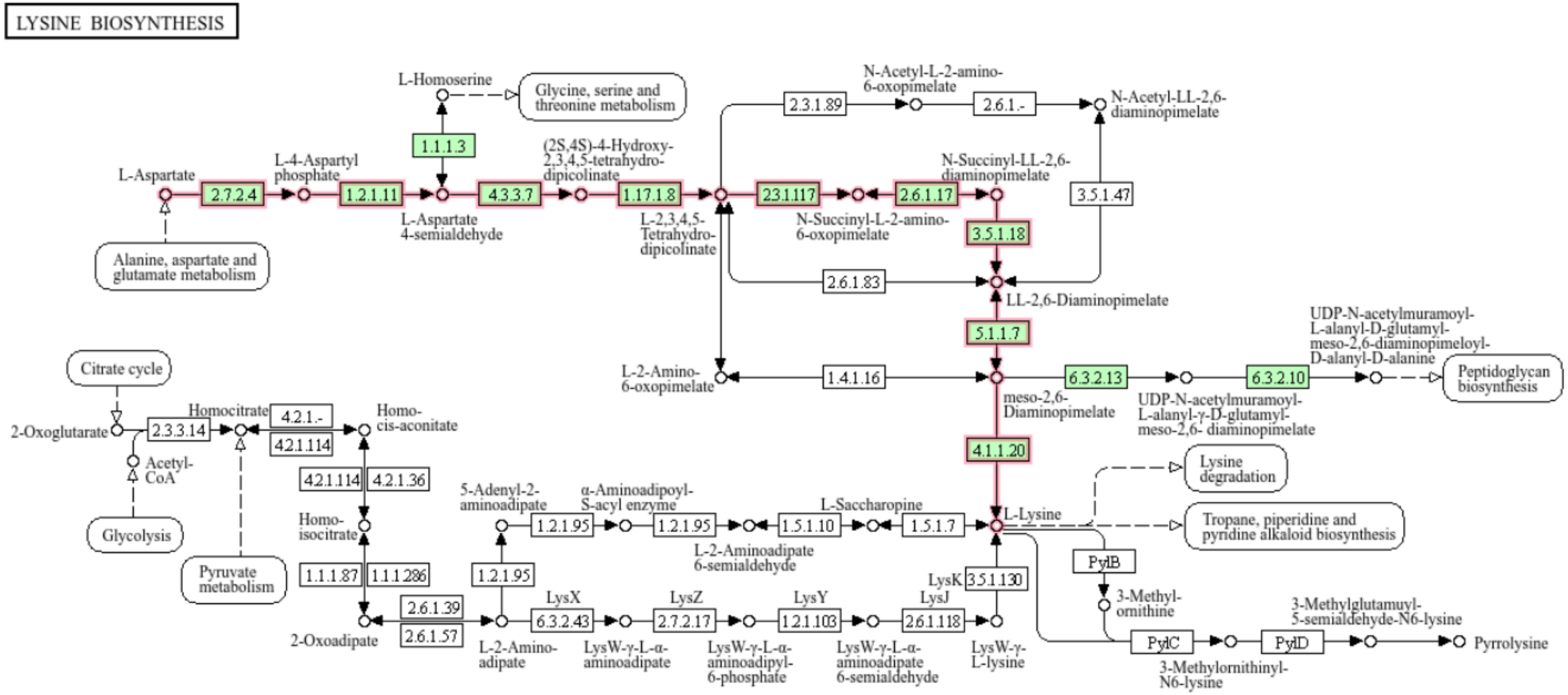

Figure S7. Biochemical pathway of enzymes and products for diaminopimelic acid synthesis encoded by the genome of strain H27-S2^{T} revealed by KEGG database (KEGG Pathway: 00300).

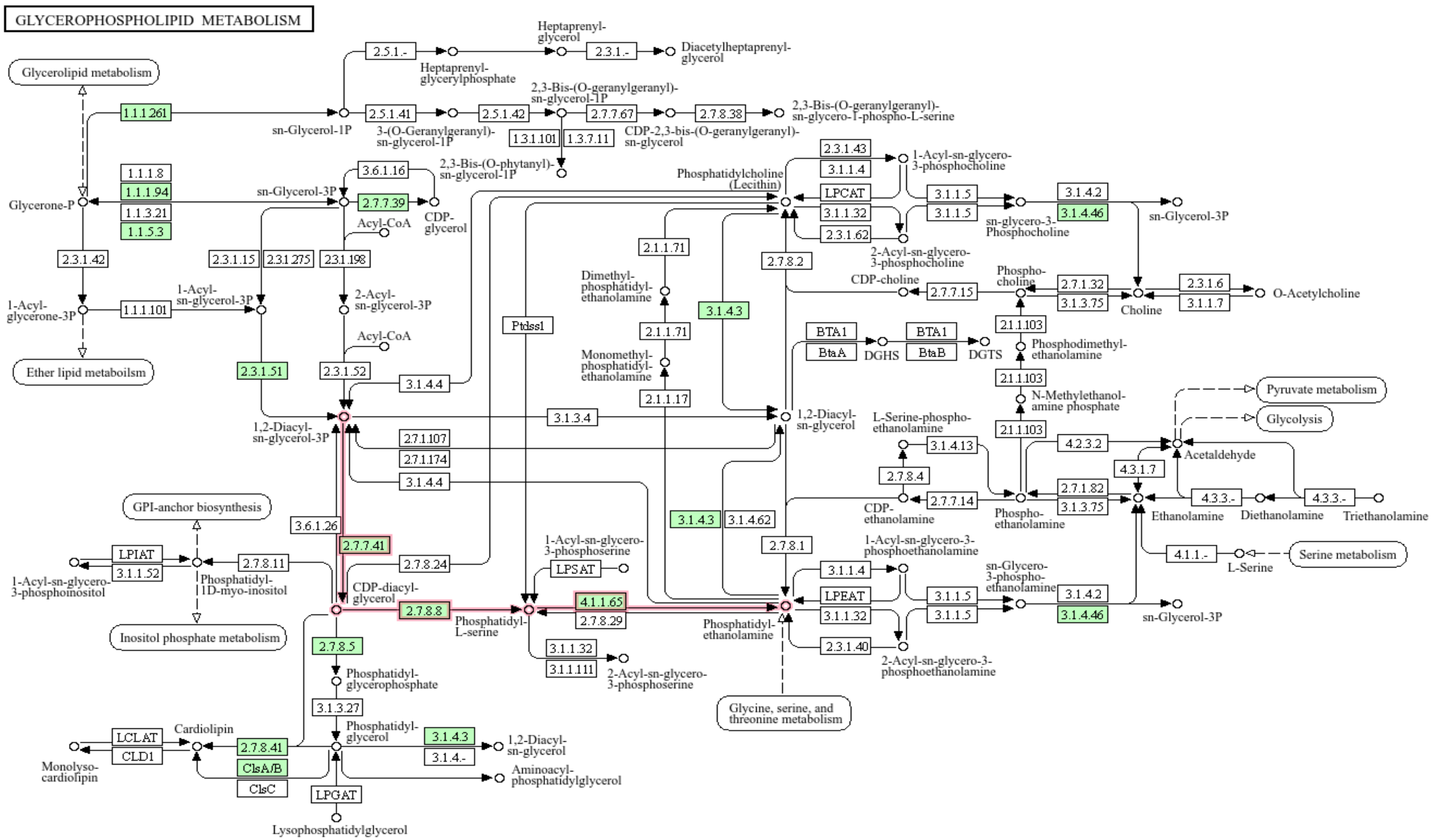

Figure S8. Biochemical pathway of enzymes and products for polar lipid synthesis encoded by the genome of strain H27-S2^{T} revealed by KEGG database (KEGG Pathway: 00564).

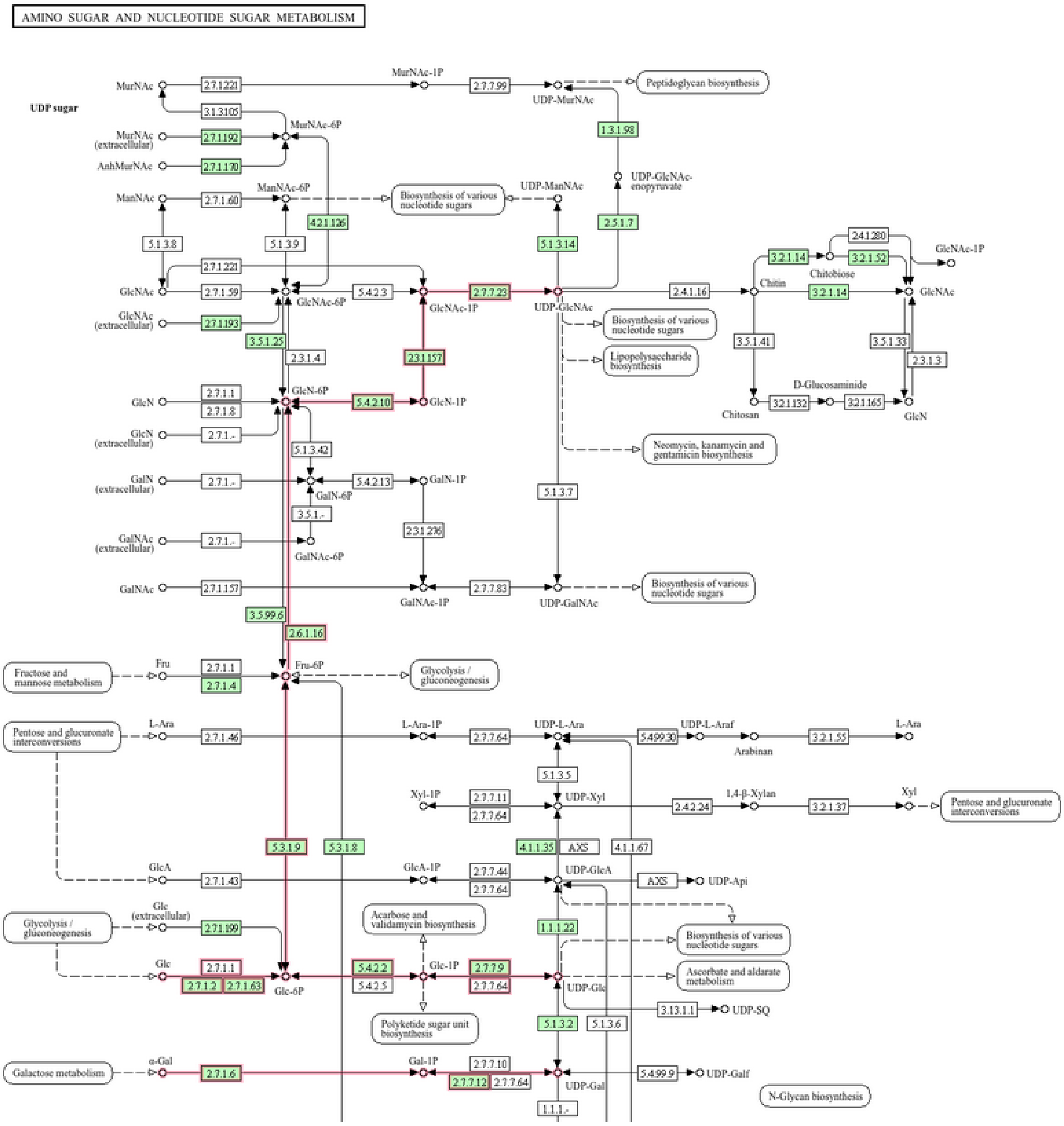

Figure S9. Biochemical pathway of enzymes and products for whole-cell sugar synthesis encoded by the genome of strain H27-S2^{T} revealed by KEGG database (KEGG Pathway: 00520).

Table S1. Differential phenotypic characteristics of strains H27-S2^T, H34-AA3, H34-S5 and closely related type strains (*Streptomyces subrutilus* ATCC 27467^T, *Streptomyces xanthophaeus* NRRL B-5414^T, and *Streptomyces avidinii* DSM 40526^T) predicted by the Protologger tool. **1**: *Streptomyces* sp. H27-S2^T, **2**: *Streptomyces* sp. H34-AA3, **3**: *Streptomyces* sp. H34-S5, **4**: *Streptomyces subrutilus* ATCC 27467^T, **5**: *Streptomyces xanthophaeus* NRRL B-5414^T, **6**: *Streptomyces avidinii* DSM 40526^T

	**1**	**2**	**3**	**4**	**5**	**6**
Cellulose utilization	-	-	-	-	+	-
Sucrose utilization	+	+	-	+	+	-
The urease cluster (alpha, beta and gamma subunits)	+	+	+	+	-	+
Sulfide and L-serine are predicted to be utilised to produce L-cysteine and acetate	+	+	+	-	-	-
Riboflavin (vitamin B2) biosynthesis	-	-	-	+	+	+
Lactose degradation	+	+	+	+	+	-
Arabinose degradation	+	+	+	-	-	-
Ribose degradation	-	+	+	+	-	-
Tyrosine degradation	-	+	+	+	-	-
Methionine degradation		+	+	+	-	-
Arginine degradation I	-	-	-	-	+	+
Arginine degradation IV	-	-	-	-	+	+
Arginine degradation V	-	+	+	-	-	-
Glyoxylate bypass	-	-	-	-	-	+
Glycerol degradation II	-	-	-	+	-	-
Pyruvate:formate lyase	-	-	-	-	+	-
Ethanol production I	-	-	-	+	+	-
Lactate production	+	+	+	+	+	-

Table S2. A pairwise distance matrix based on the 16S rRNA gene sequences of strains H27-S2^T, H34-AA3, and H34-S5 is shown. Distances and standard errors are presented in the lower-left and upper-right matrices, respectively. The pairwise distance calculations were performed using MEGA 12 v 0.10 software.

	**H27-S2^T**	**H34-AA3**	**H34-S5**
**H27-S2**		0.0000000000	0.0007622276
**H34-AA3**	0.0000000000		0.0007622276
**H34-S5**	0.0007372819	0.0007372819	

Table S3. Non-pathogenic protein families of strain H27-S2^T and their potential functions detected by the PathogenFinder.

Category	Protein	Predicted Function
Enzymes and Metabolic Proteins	Putative hydrogenase-1 large subunit	Involved in hydrogen metabolism
	Putative amidohydrolase	Likely involved in breaking down amide bonds
	Putative 3-hydroxyacyl-CoA dehydrogenase	Plays a role in fatty acid metabolism
	NADH dehydrogenase chain D / NuoB2	Part of the electron transport chain in respiration
	Isobutyryl-CoA mutase, chain B	Participates in amino acid and lipid metabolism
Regulatory and Transcription Factors	Putative transcriptional regulators (TetR-family, LacI-family, etc.)	Control gene expression, often linked to antibiotic resistance or metabolism
	RNA polymerase sigma factor, sigma L	Regulates bacterial transcription in response to environmental signals
	Putative anti-sigma factor antagonist	Influences sigma factor activity, regulating stress responses
Transport and Membrane Proteins	ABC transporter ATP-binding subunit	Part of a transport system moving molecules across membranes
	Putative integral membrane protein	Likely involved in maintaining cell structure or transport functions
Hypothetical and Conserved Proteins	Several hypothetical proteins	Their function is unknown but may play structural or enzymatic roles
	Conserved hypothetical proteins	Likely important but not yet characterized
Stress Response and Adaptation Proteins	Putative cold shock proteins	Help bacteria survive low temperatures
	Putative hydrogen peroxide-sensitive repressor	Regulates oxidative stress response
	BldD regulator	Controls bacterial differentiation and secondary metabolite production
DNA-Related Proteins	Putative Holliday junction nuclease	Involved in DNA recombination and repair
	Putative IS5 family IS1647-like transposase	Likely involved in mobile genetic elements and horizontal gene transfer
Other Functional Proteins	Putative sporulation protein	May play a role in bacterial spore formation
	Putative electron transport protein	Possibly involved in respiration or redox reactions
	Putative small secreted protein	Likely involved in bacterial communication or interaction with the environment

Table S4. Summary of the antimicrobial resistance models detected in the genomes of strains H27-S2^T, H34-AA3, and H34-S5, aligned with the ARTS server's resistance models.

**Resfam Resistance Model No**	**Description**	**Protein**	**Predicted Role**
RF0002	AAC3	Aminoglycoside acetyltransferase	Confers resistance to aminoglycosides by modifying them (Resfam)
RF0007	ABC_efflux	ABC transporter	Efflux of toxic compounds, including antibiotics (Resfam)
RF0051	Chlor_Efflux_Pump	Chloramphenicol efflux pump	Pumps out chloramphenicol, contributing to antibiotic resistance (Resfam)
RF0053	ClassA	Class A beta-lactamase	Breaks down beta-lactam antibiotics, causing resistance (Resfam)
RF0054	ClassB	Class B beta-lactamase	Breaks down beta-lactam antibiotics, causing resistance (Resfam)
RF0123	SubclassB1	Subclass B1 (metallo-) beta-lactamase	Hydrolize penicillins, cephalosporins and carbapenems, causing resistance (Resfam)
PF00044.19	Gp_dh_N	Glutamate dehydrogenase	Involved in amino acid metabolism and nitrogen balance (ARTS)
PF00185.19	OTCace	Oxytetracycline acetylase	Modifies oxytetracycline, conferring resistance (ARTS)
PF00204.20	DNA_gyraseB	DNA gyrase B	Involved in DNA replication, target for quinolone antibiotics (ARTS)
PF00227.21	Proteasome	Proteasome	Degrades damaged or unneeded proteins, regulating cellular processes (ARTS)
PF00364.17	Biotin_lipoyl	Biotin lipoate synthetase	Involved in biotin metabolism, critical for cell function (ARTS)
PF00521.15	DNA_topoisoIV	DNA topoisomerase IV	Relieves DNA supercoiling during replication (ARTS)
PF01039.17	Carboxyl_trans	Carboxyl transferase	Catalyzes the transfer of carboxyl groups, involved in metabolic processes (ARTS)
PF13599.1	Pentapeptide_4	Pentapeptide synthetase	Involved in the biosynthesis of pentapeptides, related to antibiotic resistance (ARTS)
TIGR02013	TIGR02013	DNA-directed RNA polymerase, beta subunit	Resistance due to inhibition of RNA polymerase (ARTS)
TIGR00663	TIGR00663	DNA polymerase III, beta subunit	Resistance due to interference with DNA polymerization (ARTS)

* Resfam: Prediction was carried out by ResFam codes sourced from the Dantas Lab, utilizing data available at https://dantaslab.wustl.edu/resfams/resfams_metadata.txt.

*ARTS: Function predicted by ARTS

Table S5. Gene clusters responsible for the biosynthesis of bioactive secondary metabolites in strain H27-S2^{T}, identified by the antiSMASH server and compared with the MIBiG database.

AntiSMASH				MIBIG				
**Region**	**Type**	**Most similar known cluster**	**Similarity**	**Reference**	**Similarity Score**	**Type**	**Compound(s)**	**Organism**
Region 1.1	butyrolactone	griseoviridin/fijimycin A	8%	BGC0000849	0.39	Other (Butyrolactone)	SCB1, SCB2, SCB3	*Streptomyces coelicolor* A3(2)
Region 5.1	T1PKS, hglE-KS	hexacosalactone A	9%	BGC0002007	0.55	Terpene	atolypene A, atolypene B	*Amycolatopsis tolypomycina*
Region 5.2	CDPS	mycotrienin I	7%	BGC0001845	0.05	Other (Aminocoumarin), Polyketide, NRP	alpiniamide	*Streptomyces* sp. CBMAI 2042
Region 11.1	T2PKS	gilvocarcin V	81%	BGC0000231	0.52	Polyketide	griseusin A, griseusin B	*Streptomyces griseus*
Region 11.2	CDPS	BD-12	10%	BGC0001948	0.35	Other	naseseazine C, C3-aryl pyrroloindolines	*Streptomyces* sp.
Region 12.1	other,arylpolyene	streptonigrin	7%	BGC0002362	0.47	Polyketide	loseolamycin A1, loseolamycin A2	*Micromonospora endolithica*
Region 12.2	T1PKS, butyrolactone	neocarzinostatin	54%	BGC0000849	0.7	Other (Butyrolactone)	SCB1, SCB2, SCB3	*Streptomyces coelicolor* A3(2)
Region 13.1	terpene	isorenieratene	100%	BGC0000664	0.57	Terpene	isorenieratene	*Streptomyces griseus* subsp. *griseus* NBRC 13350
Region 14.1	terpene	bombyxamycin A/bombyxamycin B	3%	BGC0002309	0.33	Terpene	cattleyene	*Streptomyces cattleya* NRRL 8057 = DSM 46488
Region 15.1	NI-siderophore	kinamycin	16%	BGC0001478	0.35	Other	desferrioxamine E	*Streptomyces* sp. ID38640
Region 17.1	NRPS-like,hydrogen-cyanide	aborycin	21%	BGC0000844	0.34	Other	clavulanic acid	*Streptomyces clavuligerus*
Region 25.1	hglE-KS, T1PKS, RiPP-like	nataxazole	7%	BGC0002578	0.57	Other	gaburedin A, gaburedin B, gaburedin C, gaburedin D, gaburedin E, gaburedin F	*Streptomyces venezuelae* ATCC 10712
Region 28.1	NRPS	skyllamycin A/skyllamycin B	8%	BGC0002071	0.4	NRP	virginiafactin A, virginiafactin B, virginiafactin C, virginiafactin D	*Pseudomonas* sp. QS1027
Region 33.1	NRPS-like,NRPS,lanthipeptide-class-iii	JBIR-126	96%	BGC0000551	**1.12**	RiPP	SapB	*Streptomyces coelicolor* A3(2)
Region 34.1	terpene	saframycin A/saframycin B	4%	BGC0001911	0.36	Terpene	raimonol	*Streptomyces anulatus*
Region 34.2	ectoine	kosinostatin	9%	BGC0000859	0.27	Other	ectoine	*Methylomicrobium alcaliphilum*
Region 44.1	melanin	melanin	100%	BGC0000911	0.54	Other	melanin	*Streptomyces* griseus subsp. *griseus* NBRC 13350
Region 44.2	NI-siderophore, NRPS-like,T1PKS	miharamycin B/miharamycin A	92%	BGC0001956	**1.2**	Polyketide	miharamycin A, miharamycin B	*Streptomyces miharaensis*
Region 44.3	T3PKS	alkylresorcinol	100%	BGC0000282	0.55	Polyketide	alkylresorcinol	*Streptomyces griseus* subsp. *griseus* NBRC 13350
Region 44.4	butyrolactone			BGC0000848	0.37	Other	A-factor	*Streptomyces griseus* subsp. *griseus* NBRC 13350
Region 49.1	T2PKS	spore pigment	66%	BGC0000272	0.43	Polyketide	spore pigment	*Streptomyces collinus*

Table S5. Gene clusters responsible for the biosynthesis of bioactive secondary metabolites in strain H27-S2^{T}, identified by the antiSMASH server and compared with the MIBiG database (continued).

AntiSMASH				MIBIG				
**Region**	**Type**	**Most similar known cluster**	**Similarity**	**Reference**	**Similarity Score**	**Type**	**Compound(s)**	**Organism**
Region 55.1	NRP-metallophore,NRPS	peucechelin	55%	BGC0002424	**1.03**	NRP	saccharochelin A, saccharochelin B, saccharochelin C, saccharochelin D, saccharochelin E, saccharochelin F, saccharochelin G, saccharochelin H	*Saccharothrix* sp.
Region 55.2	indole	5-isoprenylindole-3-carboxylate β-D-glycosyl ester	23%	BGC0001294	0.37	Other	7-prenylisatin	*Streptomyces* sp. MBT28
Region 55.3	NAPAA	ε-Poly-L-lysine	100%	BGC0002174	0.39	NRP	ε-Poly-L-lysine	*Epichloe festucae*
Region 66.1	RiPP-like			BGC0001486	0.09	RiPP	3-thiaglutamate	*Pseudomonas syringae* pv. *maculicola* str. ES4326
Region 66.2	terpene	toxoflavin/fervenulin	14%	BGC0001181	0.42	Terpene	geosmin	*Streptomyces coelicolor* A3(2)
Region 66.3	T1PKS	4-Z-annimycin	77%	BGC0001298	0.48	Polyketide	4-Z-annimycin	*Streptomyces calvus*
Region 71.1	butyrolactone			BGC0000848	0.33	Other	A-factor	*Streptomyces griseus* subsp. *griseus* NBRC 13350
Region 77.1	NRPS-like,NRPS,NRP-metallophore	netropsin	72%	BGC0000375	**1.36**	NRP	indigoidine	*Streptomyces chromofuscus*
Region 77.2	NRPS,NRPS-like,NAPAA	antipain	100%	BGC0002051	**1.37**	NRP	antipain	*Streptomyces* sp
Region 77.3	amglyccycl,terpene	streptomycin	55%	BGC0000658	0.88	Terpene	2-methylisoborneol	*Streptomyces griseus* subsp. *griseus* NBRC 13350
Region 82.1	terpene	hopene	61%	BGC0000663	0.34	Terpene	hopene	*Streptomyces coelicolor* A3(2)

* The highlighted similarity values in green signify a substantial and meaningful resemblance between the corresponding biosynthetic gene clusters and the well-known gene clusters associated with secondary metabolites. In contrast, the similarity values marked in orange indicate a lower degree of similarity. Values that are not marked suggest a lack of similarity. Similarities with high scores in the MIBiG database are highlighted in bold.

Table S6. Gene clusters responsible for the biosynthesis of bioactive secondary metabolites in strain H34-AA3, identified by the antiSMASH server and compared with the MIBiG database.

AntiSMASH				MIBIG				
**Region**	**Type**	**Most similar known cluster**	**Similarity**	**Reference**	**Similarity Score**	**Type**	**Compound(s)**	**Organism**
Region 11.1	lanthipeptide-class-iii,NRPS,NRPS-like	JBIR-126	96%	BGC0000551	**1.12**	RiPP	SapB	*Streptomyces coelicolor* A3(2)
Region 30.1	butyrolactone	lactonamycin	3%	BGC0000848	0.33	Other	A-factor	*Streptomyces griseus* subsp. *griseus* NBRC 13350
Region 57.1	T1PKS,butyrolactone	neocarzinostatin	54%	BGC0000849	0.7	Other (Butyrolactone)	SCB1, SCB2, SCB3	*Streptomyces coelicolor* A3(2)
Region 57.2	arylpolyene,other	streptonigrin	7%	BGC0002362	0.47	Polyketide	loseolamycin A1, loseolamycin A2	*Micromonospora endolithica*
Region 59.1	NRP-metallophore,NRPS,RiPP-like	peucechelin	55%	BGC0002424	**1.03**	NRP	saccharochelin A, saccharochelin B, saccharochelin C, saccharochelin D, saccharochelin E, saccharochelin F, saccharochelin G, saccharochelin H	*Saccharothrix* sp.
Region 59.2	indole	5-isoprenylindole-3-carboxylate β-D-glycosyl ester	23%	BGC0001294	0.37	Other	7-prenylisatin	*Streptomyces* sp. MBT28
Region 59.3	NAPAA	ε-Poly-L-lysine	100%	BGC0002174	0.39	NRP	ε-Poly-L-lysine	*Epichloe festucae*
Region 60.1	terpene	bombyxamycin A/bombyxamycin B	3%	BGC0002309	0.33	Terpene	cattleyene	*Streptomyces cattleya* NRRL 8057 = DSM 46488
Region 65.1	T2PKS	spore pigment	66%	BGC0000272	0.43	Polyketide	spore pigment	*Streptomyces collinus*
Region 68.1	NRPS-like,NRPS	netropsin	72%	BGC0000375	**1.03**	NRP	indigoidine	*Streptomyces chromofuscus*
Region 69.1	NI-siderophore	kinamycin	16%	BGC0001478	0.35	Other	desferrioxamine E	*Streptomyces* sp. ID38640
Region 70.1	crocagin	massinidine	66%	BGC0000820	0.34	Alkaloid	physostigmine	*Streptomyces griseofuscus*
Region 71.1	NRPS	skyllamycin A/skyllamycin B	8%	BGC0002017	0.36	NRP	potensibactin	*Nocardiopsis potens* DSM 45234
Region 81.1	terpene	hopene	61%	BGC0000663	0.34	Terpene	hopene	*Streptomyces coelicolor* A3(2)
Region 81.2	ectoine	ectoine	100%	BGC0000853	0.6	Other	ectoine	*Streptomyces anulatus*
Region 85.1	terpene,amglyccycl	streptomycin	55%	BGC0000658	**0.88**	Terpene	2-methylisoborneol	*Streptomyces griseus* subsp. *griseus* NBRC 13350
Region 89.1	NAPAA,NRPS-like,NRPS	antipain	100%	BGC0002051	**1.44**	NRP	antipain	*Streptomyces* sp.
Region 91.1	butyrolactone	griseoviridin/fijimycin A	8%	BGC0000849	0.39	Other (Butyrolactone)	SCB1, SCB2, SCB3	*Streptomyces coelicolor* A3(2)
Region 95.1	terpene	isorenieratene	100%	BGC0000664	0.57	Terpene	isorenieratene	*Streptomyces griseus* subsp. *griseus* NBRC 13350
Region 103.1	hydrogen-cyanide,NRPS-like	aborycin	21%	BGC0000844	0.34	Other	clavulanic acid	*Streptomyces clavuligerus*
Region 103.2	RiPP-like			BGC0001486	0.09	RiPP	3-thiaglutamate	*Pseudomonas syringae* pv. *maculicola* str. ES4326
Region 103.3	terpene	toxoflavin/fervenulin	14%	BGC0001181	0.42	Terpene	geosmin	*Streptomyces coelicolor* A3(2)
Region 114.1	NI-siderophore,NRPS-like,T1PKS	miharamycin B/miharamycin A	92%	BGC0001956	**1.2**	Polyketide	miharamycin A, miharamycin B	*Streptomyces miharaensis*

Table S6. Gene clusters responsible for the biosynthesis of bioactive secondary metabolites in strain H34-AA3, identified by the antiSMASH server and compared with the MIBiG database (continued).

AntiSMASH				MIBIG				
Region	Type	Most similar known cluster	Similarity	Reference	Similarity Score	Type	Compound(s)	Organism
Region 114.2	T3PKS	alkylresorcinol	100%	BGC0000282	0.55	Polyketide	alkylresorcinol	*Streptomyces griseus* subsp. *griseus* NBRC 13350
Region 124.1	melanin	istamycin	4%	BGC0000911	0.54	Other	melanin	*Streptomyces griseus* subsp. *griseus* NBRC 13350
Region 125.1	CDPS	BD-12	10%	BGC0001948	0.35	Other	naseseazine C, C3-aryl pyrroloindolines	*Streptomyces* sp.
Region 126.1	terpene	saframycin A/saframycin B	6%	BGC0001911	0.36	Terpene	raimonol	*Streptomyces anulatus*
Region 136.1	NRPS,T1PKS,PKS-like	maklamicin	10%	BGC0000016	**1.1**	Other	amphotericin B	*Streptomyces nodosus*
Region 143.1	ectoine	kosinostatin	9%	BGC0000859	0.27	Other	ectoine	*Methylomicrobium alcaliphilum*

* The highlighted similarity values in green signify a substantial and meaningful resemblance between the corresponding biosynthetic gene clusters and the well-known gene clusters associated with secondary metabolites. In contrast, the similarity values marked in orange indicate a lower degree of similarity. Values that are not marked suggest a lack of similarity. Similarities with high scores in the MIBiG database are highlighted in bold.

Table S7. Gene clusters responsible for the biosynthesis of bioactive secondary metabolites in strain H34-S5, identified by the antiSMASH server and compared with the MIBiG database.

AntiSMASH				MIBIG				
**Region**	**Type**	**Most similar known cluster**	**Similarity**	**Reference**	**Similarity Score**	**Type**	**Compound(s)**	**Organism**
Region 3.1	NRP-metallophore,NRPS	peucechelin	25%	BGC0001133	**0.81**	NRP	taxlllaid A	*Xenorhabdus bovienii* SS-2004
Region 14.1	melanin	melanin	100%	BGC0000911	0.54	Other	melanin	*Streptomyces griseus* subsp. *griseus* NBRC 13350
Region 17.1	RiPP-like							
Region 25.1	butyrolactone	neocarzinostatin	4%	BGC0000848	0.34	Other	A-factor	*Streptomyces griseus* subsp. *griseus* NBRC 13350
Region 35.1	NRPS			BGC0001135	0.34	NRP	bicornutin A1, bicornutin A2	*Xenorhabdus budapestensis*
Region 39.1	terpene	toxoflavin/fervenulin	14%	BGC0001181	0.42	Terpene	geosmin	*Streptomyces coelicolor* A3(2)
Region 46.1	NRPS-like	netropsin	9%	BGC0000900	0.32	Other	ferrichrome	*Aspergillus oryzae*
Region 55.1	ectoine	ectoine	100%	BGC0000853	0.6	Other	ectoine	*Streptomyces anulatus*
Region 58.1	ectoine	kosinostatin	9%	BGC0000859	0.27	Other	ectoine	*Methylomicrobium alcaliphilum*
Region 62.1	NRPS	cinnapeptin	10%	BGC0002075	0.33	NRP, Alkaloid	pyreudione A, pyreudione B, pyreudione C, pyreudione D, pyreudione E	*Pseudomonas fluorescens*
Region 122.1	NRPS-like	aborycin	14%	BGC0000844	0.34	Other	clavulanic acid	*Streptomyces clavuligerus*
Region 133.1	other,arylpolyene	streptonigrin	7%	BGC0002362	0.47	Polyketide	loseolamycin A1, loseolamycin A2	*Micromonospora endolithica*
Region 133.2	T1PKS	neocarzinostatin	45%	BGC0000056	0.37	Polyketide	esperamicin	*Actinomadura verrucosospora*
Region 146.1	butyrolactone			BGC0000848	0.33	Other	A-factor	*Streptomyces griseus* subsp. *griseus* NBRC 13350
Region 155.1	butyrolactone	griseoviridin/fijimycin A	8%	BGC0000849	0.39	Other (Butyrolactone)	SCB1, SCB2, SCB3	*Streptomyces coelicolor* A3(2)
Region 199.1	terpene,amglyccycl	streptomycin	55%	BGC0000658	**0.88**	Terpene	2-methylisoborneol	*Streptomyces griseus* subsp. *griseus* NBRC 13350
Region 204.1	crocagin	massinidine	66%	BGC0000820	0.34	Alkaloid	physostigmine	*Streptomyces griseofuscus*
Region 205.1	NI-siderophore	kinamycin	16%	BGC0001478	0.35	Other	desferrioxamine E	*Streptomyces* sp. ID38640
Region 206.1	terpene	hopene	61%	BGC0000663	0.34	Terpene	hopene	*Streptomyces coelicolor* A3(2)
Region 208.1	NRPS,NRPS-like,NAPAA	antipain	100%	BGC0002051	**1.44**	NRP	antipain	*Streptomyces* sp.
Region 210.1	T3PKS	alkylresorcinol	100%	BGC0000282	0.55	Polyketide	alkylresorcinol	*Streptomyces griseus* subsp. *griseus* NBRC 13350

Table S7. Gene clusters responsible for the biosynthesis of bioactive secondary metabolites in strain H34-S5, identified by the antiSMASH server and compared with the MIBiG database (continued).

AntiSMASH				MIBIG				
**Region**	**Type**	**Most similar known cluster**	**Similarity**	**Reference**	**Similarity Score**	**Type**	**Compound(s)**	**Organism**
Region 215.1	NRPS,RiPP-like			BGC0001485	0.43	Alkaloid	chuangxinmycin	*Actinoplanes tsinanensis*
Region 221.1	NRPS	leinamycin	4%	BGC0000375	0.35	NRP	indigoidine	*Streptomyces chromofuscus*
Region 226.1	terpene	isorenieratene	100%	BGC0000664	0.57	Terpene	isorenieratene	*Streptomyces griseus* subsp. *griseus* NBRC 13350
Region 229.1	NRPS	7-deoxypactamycin	13%	BGC0000343	0.32	NRP	enterobactin	*Pseudomonas* sp. J465
Region 232.1	terpene	bombyxamycin A/bombyxamycin B	3%	BGC0002309	0.33	Terpene	cattleyene	*Streptomyces cattleya* NRRL 8057 = DSM 46488
Region 240.1	NRPS-like,T1PKS,NI-siderophore	miharamycin B/miharamycin A	85%	BGC0001956	**1.11**	Polyketide	miharamycin A, miharamycin B	*Streptomyces miharaensis*
Region 243.1	T2PKS	spore pigment	66%	BGC0000272	0.43	Polyketide	spore pigment	*Streptomyces collinus*
Region 251.1	CDPS	BD-12	10%	BGC0001948	0.35	Other	naseseazine C, C3-aryl pyrroloindolines	*Streptomyces* sp.
Region 253.1	NRPS,PKS-like,T1PKS	everninomicin A	6%	BGC0000016	**1.1**	Other	amphotericin B	*Streptomyces nodosus*
Region 259.1	NRPS-like,NRPS,lanthipeptide-class-iii	JBIR-126	96%	BGC0000551	**1.11**	RiPP	SapB	*Streptomyces coelicolor* A3(2)
Region 260.1	indole	5-isoprenylindole-3-carboxylate β-D-glycosyl ester	23%	BGC0000622	0.32	RiPP	megacin	*Bacillus megaterium*
Region 266.1	terpene	saframycin A/saframycin B	6%	BGC0001911	0.36	Terpene	raimonol	*Streptomyces anulatus*
Region 272.1	NRPS,NRPS-like	lipopeptide 8D1-1/lipopeptide 8D1-2	4%	BGC0001050	**0.78**	NRP, Polyketide	thalassospiramide A	*Tistrella bauzanensis*
Region 276.1	NRPS-like,NRPS	netropsin	50%	BGC0000343	0.62	NRP	enterobactin	*Pseudomonas* sp. J465

* The highlighted similarity values in green signify a substantial and meaningful resemblance between the corresponding biosynthetic gene clusters and the well-known gene clusters associated with secondary metabolites. In contrast, the similarity values marked in orange indicate a lower degree of similarity. Values that are not marked suggest a lack of similarity. Similarities with high scores in the MIBiG database are highlighted in bold.

Table S8. Presence of genomic features related to resistance to antibiotics and toxic compounds in the genomes of strains H27-S2^T, H34-AA3 and H34-S5, revealed by RAST web server.

Resistance Mechanism	H27-S2^T	H34-AA3	H34-S5
Arsenic resistance	+	+	+
Beta-lactamase activity	+	+	+
Chloramphenicol resistance	+	+	+
Cobalt-zinc-cadmium resistance	+	+	+
Copper homeostasis	+	+	+
Copper homeostasis, copper tolerance	+	+	+
Daunorubicin/doxorubicin resistance	-	+	+
Ethidium bromide methyl viologen resistance	+	+	+
Fluoroquinolone resistance	+	+	+
Glioxalase/bleomycin resistance	-	+	+
Mercury reductase	+	+	+
Multidrug resistance	+	+	+
Organic hydroperoxide resistance	+	+	+
Oxytetracycline resistance	+	-	+
Resistance to chromium compounds	+	+	+
Tellurite resistance	-	-	+
Tellurium resistance	+	+	+
Tunicamycin resistance	+	-	-
